# Inhibition of Neural Crest Cell Migration by Strobilurin Fungicides and Other Mitochondrial Toxicants

**DOI:** 10.3390/cells13242057

**Published:** 2024-12-12

**Authors:** Viktoria Magel, Jonathan Blum, Xenia Dolde, Heidrun Leisner, Karin Grillberger, Hiba Khalidi, Iain Gardner, Gerhard F. Ecker, Giorgia Pallocca, Nadine Dreser, Marcel Leist

**Affiliations:** 1 In Vitro Toxicology and Biomedicine, Dept Inaugurated by the Doerenkamp-Zbinden Foundation, University of Konstanz, 78464 Konstanz, Germany; 2 Department of Pharmaceutical Chemistry, University of Vienna, 1090 Vienna, Austria; 3 Certara Predictive Technologies, Level 2-Acero, 1 Concourse Way, Sheffield S1 2BJ, UK; 4 Center for Alternatives to Animal Testing in Europe (CAAT-Europe), University of Konstanz, 78464 Konstanz, Germany

**Keywords:** neural crest cells, mitochondria, developmental toxicity, strobilurin fungicides, next-generation risk assessment, toxicokinetics, hit confirmation, adverse outcome pathway, data integration

## Abstract

Cell-based test methods with a phenotypic readout are frequently used for toxicity screening. However, guidance on how to validate the hits and how to integrate this information with other data for purposes of risk assessment is missing. We present here such a procedure and exemplify it with a case study on neural crest cell (NCC)-based developmental toxicity of picoxystrobin. A library of potential environmental toxicants was screened in the UKN2 assay, which simultaneously measures migration and cytotoxicity in NCC. Several strobilurin fungicides, known as inhibitors of the mitochondrial respiratory chain complex III, emerged as specific hits. From these, picoxystrobin was chosen to exemplify a roadmap leading from cell-based testing towards toxicological predictions. Following a stringent confirmatory testing, an adverse outcome pathway was developed to provide a testable toxicity hypothesis. Mechanistic studies showed that the oxygen consumption rate was inhibited at sub-µM picoxystrobin concentrations after a 24 h pre-exposure. Migration was inhibited in the 100 nM range, under assay conditions forcing cells to rely on mitochondria. Biokinetic modeling was used to predict intracellular concentrations. Assuming an oral intake of picoxystrobin, consistent with the acceptable daily intake level, physiologically based kinetic modeling suggested that brain concentrations of 0.1–1 µM may be reached. Using this broad array of hazard and toxicokinetics data, we calculated a margin of exposure ≥ 80 between the lowest in vitro point of departure and the highest predicted tissue concentration. Thus, our study exemplifies a hit follow-up strategy and contributes to paving the way to next-generation risk assessment.

## 1. Introduction

Disturbances of neural crest cell (NCC) differentiation and function can have serious consequences on the development of vertebrates. As this is a fetal cell population, its dysfunction in humans can only be studied retrospectively or indirectly. One approach is to differentiate such cells from human induced pluripotent stem cells (hiPSCs). This approach has led to the development of toxicological tests using human NCCs.

During fetal development, the neural crest is formed by epithelial-to-mesenchymal transition (EMT) of the tissue at the border of the neural plate [1]. The cells migrate long distances through the body to form certain smooth muscle cells, craniofacial structures, skin melanocytes, the adrenal medulla, and parts of the nervous system [2,3]. Several chemicals are known to adversely affect the NCCs, and genetic defects that impair the localization of these cells cause serious malformations, such as Hirschsprung’s disease [4,5]. Therefore, chemicals that inhibit the migration of NCCs to their target location need to be considered as potential developmental toxicants. Exposure to such substances may cause craniofacial malformations, neural tube closure defects, or other features of developmental neurotoxicity (DNT). To identify chemicals with this potential hazard, an NCC function assay has been developed [6], applied in various screens and incorporated in the DNT in vitro battery (IVB) assembled by the European Food Safety Authority (EFSA) and an Organisation of Economic Cooperation and Development (OECD) working group [7,8,9]. This test measures the inhibition of the migration of NCCs into a circular area (cMINC). Several compounds suspected to have a DNT hazard for humans (e.g., cadmium, methyl mercury, brominated diphenyl ethers) [10,11,12] are hits (migration inhibitors) in the cMINC assay [6,7,8].

NCC migration is a fundamental neurodevelopmental process [10,13]. Rather than being a single “mechanism”, it involves several signaling functions and biochemical processes. Accordingly, compounds with different targets, modes of action and associations to adverse outcome pathways (AOPs) may be detected. Examples include the actin polymerization inhibitor cytochalasin D, the tubulin stabilizer taxol, gap-junction-modulating non-planar PCBs, the mitochondrial respiratory chain complex I (cI) inhibitor rotenone or some triazole pesticides [7,14]. Also, various chemoattractants (migration enhancers) are known (e.g., human serum) that may disturb the normal migration pattern [15].

Human neurodevelopment entails intricate biological mechanisms. Chemical exposure might detrimentally affect them and thus lead to lasting neurological deficiencies. The surge in documented neurodevelopmental impairments among infants and children is thought to stem, in part, from exposure to specific chemicals before and after birth [11,12]. Traditional DNT testing methods, such as the OECD test guideline 443 (TG443) [16] study with a developmental neurotoxicity cohort, provide valuable data on neurodevelopmental outcomes. Since such tests are only rarely applied, there is a need for more scalable alternatives. To overcome the throughput limitations tied to conventional (in vivo) DNT testing protocols, there has been an increased emphasis on new approach methods (NAMs) [17]. Thus, NAM-based screening is particularly valuable to help prioritize the vast number of compounds lacking DNT information for further testing. In the future, a combination of various NAMs in an in vitro test battery, toxicokinetic modeling, and in vitro-to-in vivo extrapolation approaches may fill the toxicological information gap for certain groups of chemicals [8,18,19,20,21].

Various screenings have been performed in the DNT field. Some have used several assays to provide complementary information, e.g., on small libraries (80–120 compounds) assembled by the US NTP [7,20,22], EFSA [8] or the US Centre of Computational Toxicology [18]. Only very few screenings involved larger libraries [23], and the application to environmental samples is still rare [24]. Some additional screening experience has been gained from related fields, e.g., neurotoxicity [25] or developmental toxicity [26,27,28,29].

Screening for hazardous properties is still less common than for biochemical targets or pharmacological effects. One of the reasons may be that the steps following a screen are better defined and motivated for drug discovery. A procedure for hit follow-up is not clearly established in toxicology. The value of toxicological screen hits has already largely increased by the systematic incorporation of concentration-dependency as an output parameter [30,31] and by using the NAM data for in vivo estimated adverse dose and safety margin predictions [32]. Another approach that helps to contextualize screen data is to relate them to known human (or animal) blood levels. Examples can be found in some developmental toxicity screens [33,34].

The classical drug discovery approach to screen hits is to repeat the primary assay under non-screen conditions (new verified stocks, densely spaced concentrations, stringent requirements for test outcomes of hit and accompanying controls). The next steps then involve confirmatory (also called bio-orthogonal testing) assays that address the same target but use other test systems, endpoints, and overall setups. This procedure eliminates many technical artefacts and increases confidence into a broader biological validity of hits. In toxicology, whole fields are devoted to such questions. They include investigative toxicology for industry applications [35] and the large field of mechanistic toxicology in academia. Such work sometimes involves hundreds of publications on a single compound, as it needs to evaluate in a quantitative way how relevant various tests and assay conditions are for the prediction of human hazards. One way to sort and categorize such literature is to structure it along an AOP [36,37,38]. An important gap in this process is sometimes the lack of knowledge on the mode of action of a toxicant. This makes ridging from a screen exercise to providing a strong mechanistic rationale (or association to an AOP) difficult. This gap is particularly evident for hits coming from phenotypic screens. In such cases, the lack of a defined screen target provides a large threshold for follow-up studies. Only few examples show how this gap may be bridged. One proposed strategy for hit confirmation in initial screening is to validate the hit by repeating the first assay, confirm the hit in an orthogonal assay, and then elucidate a target [7].

The current study was undertaken to provide a further exemplification of the hit follow-up process. After screening a library of 115 compounds, provided by the National Institute of Environmental Health Sciences (NIEHS), hits were confirmed in an orthogonal migration assay. As the compounds were already relatively data-rich, it was possible to group the hits according to known targets. We focused on inhibitors of mitochondrial respiratory chain complex III (cIII) as they showed a high abundance of positive hits. We explored whether this mode of action would be confirmed in the NCC test system, and we selected picoxystrobin for a case study to relate the in vitro point of departure to a potential human exposure. Finally, we explored whether the information gained could be generalized by establishment of a preliminary AOP that links cIII inhibitors to developmental toxicity as an adverse outcome.

## 2. Materials and Methods

### 2.1. Neural Crest Cell Differentiation

NCCs were differentiated from the human induced pluripotent stem cell (hiPSC) line IMR90_clone_#4 (WiCell, Madison, WI, USA) following the modified protocol of Mica et al. [39] and according to Dolde et al. [14]. For the maintenance, the hiPSCs were plated on human laminin (Sigma, Steinheim, Germany) coating in essential 8 (E8) medium (DMEM/F12 supplemented with 15 mM Hepes (Gibco/Fisher Scientific, Hampton, NH, USA) supplemented with 16 mg/mL L-ascorbic-acid, 0.7 mg/mL sodium selenite, 20 μg/mL insulin, 10 μg/mL holo-transferrin (all from Sigma, Steinheim, Germany), 100 ng/mL bFGF (Thermo Fisher Scientific, Waltham, MA, USA) and 1.74 ng/mL TGFβ (R&D Systems, Minneapolis, MN, USA). For the differentiation of the hiPSCs into NCCs, the cells were replated on Matrigel^TM^ (Corning, Glendale, AZ, USA)-coated 6-well plates at a density of 100,000 cells/cm^2^ in E8 medium supplemented with 10 μM ROCK-inhibitor (Y-27632 (Tocris, Bristol, UK)). After one day, the differentiation was initiated (day of differentiation DoD 0) by a medium change to KSR medium (DMEM containing 15% knock out serum replacement (KSR), 1% GlutaMax, 1% MEM NEAA solution and 50 μM 2-mercaptoethanol (all from Gibco/Fisher Scientific, Hampton, NH, USA)), which was supplemented with 10 μM SB431542 (Tocris, Bristol, UK) and 20 ng/mL Noggin (R&D Systems, Minneapolis, MN, USA). On DoD 2, the cells were treated with 3 μM CHIR 99021 (Axon Medchem, Reston, VA, USA). On DoD 3 and 4, noggin and SB431542 were withdrawn, and on DoD 4, the medium was gradually replaced in 25% increments with N2-S medium (DMEM/F12, 1% GlutaMax (both from Gibco/Fisher Scientific, Hampton, NH, USA), 1.55 mg/mL glucose, 0.1 mg/mL apotransferin, 25 μg/mL insulin, 20 nM progesterone, 100 μM putrescine, and 30 nM selenium (all from Sigma, Steinheim, Germany)). Cells were collected on DoD 11 and resuspended in N2-S medium containing 20 ng/mL EGF and 20 ng/mL FGF2 (both from R&D Systems, Minneapolis, MN, USA). They were seeded on poly-L-ornithine (PLO)/laminin/fibronectin (all from Sigma, Steinheim, Germany) coated 10 cm dishes as droplets of 10 μL. For the expansion of the cells, splitting was performed weekly, and the N2-S medium, supplemented with 20 ng/mL EGF and FGF2, was exchanged every second day. Cells were cryopreserved after 35–39 days at a concentration of 4 × 10^6^ cells/mL in 90% N2-S medium and 10% DMSO (Merck Millipore, Burlington, MA, USA).

### 2.2. Cell Culture Handling

For every experiment, a new batch of NCCs was freshly thawed. They were cultured and seeded on poly-L-ornithine (PLO)/fibronectin/laminin-coated plates. For this, plates were coated at least two days prior to the experiments with 10 μg/mL PLO in phosphate-buffered saline (DPBS (Gibco/Fisher Scientific, Hampton, NH, USA)) and incubated overnight at 37 °C and 5% CO_2_. On the next day, the solution was aspirated, and the wells were washed twice with DPBS. Afterwards, they were additionally coated with 1 μg/mL laminin and 1 μg/mL fibronectin in DPBS. The plates were incubated at least overnight at 37 °C and 5% CO_2_ and stored for a maximum of two weeks. The solution was aspirated and air-dried within a sterile bench prior to using the plates. Seeded cells were always incubated under humidified conditions at 37 °C and 5% CO_2_, and cell culture work was carried out under sterile conditions. NCCs were always seeded in N2-S medium supplemented with 20 μg/mL of the cytokines EGF and FGF2.

### 2.3. Compound Handling

All compounds were provided by NIEHS in individual polypropylene vials (1.4 mL screw-cap tubes with silicon o-ring, 96-vial plate rack) as 100 mM stock solutions, with each vial containing 1000 μL. Argon gas headspace was added to compound vials prior to storage/shipment. The compounds were diluted in dimethyl sulfoxide (DMSO) or water, based on solubility, and stored as aliquots of 5 μL at −20 °C. Cytochalasin D (CytoD) (Sigma, Steinheim, Germany) was used as a positive control with a concentration of 200 nM and as an acceptance criteria of a successful run in the cMINC assay and the transwell assay.

### 2.4. Circular Migration of Neural Crest Cells (cMINC) Assay

The research group initially developed the first version of the test in 2012 [40]. The test has been further improved and characterized [6]. To perform the cMINC assay, NCCs were thawed in N2-S Medium and seeded on the day of migration-1 (DoM-1) on a 96-well polystyrene plate (Corning, Glendale, AZ, USA) around stoppers, to create a cell-free area of 2 mm diameter (Platypus Technologies, Madison, WI, USA). The cells were resuspended in N2-S Medium supplemented with 20 μg/mL of the cytokines EGF and FGF, and then counted and seeded around the stoppers at a density of 95,000 cells/cm^2^. After 24 h, the stoppers were removed, and the medium was exchanged. The removal of the stoppers initiates migration of the NCCs into the cell-free area. For the treatment, the 5x-concentrated toxicant solution was added to the medium 24 h after stopper removal.

On day 2, cells were stained with 533 nM calcein-AM and 1 μg/mL H-33342 (both from Sigma, Steinheim, Germany) and observed using two different channels on the Cellomics ArrayScan VTI imaging microscope (Thermo Fisher, Pittsburgh, PA, USA) to assess viability and migration. For the migration, four pictures were taken in the region of interest (ROI) (5× magnification objective). Migration data were obtained using the software “Ringassay” (http://invitrotox.uni-konstanz.de/ (accessed on 3 November 2024)), which can estimate the previously cell-free area and count the number of H-33342 and calcein-AM double-positive cells. Cell viability was assessed by taking four pictures outside of the ROI with 10× magnification objective. Viable cells were defined as H-33342 and calcein-AM double-positive cells and calculated by an automatic algorithm of the ArrayScan VTI 700 Series software as described earlier [6,41]. When the cMINC assay was used in a high-throughput screening approach, all plates had a standardized layout. Each plate included wells for testing of negative (0.1% DMSO solvent) and positive controls (200 nM cytochalasin D) in addition to the wells for blinded samples. The data from control wells were used as acceptance criteria of the assay. A run was rejected if treatment with CytoD did not inhibit migration by at least 25% and when cell viability in negative controls was below 90%. Renormalization of the concentration–response curves was performed after a visual inspection that the two lowest test-concentration data points are in the no-effect range. The average response (relative to solvent control) of the lowest two test concentrations was calculated, and all other data points of the same curve were divided by this average value. The calculations of the benchmark concentrations 10 for viability (BMC_10_ (V)) and 25 for migration (BMC_25_ (M)) were performed with the online available BMC software (http://invitrotox.uni-konstanz.de/ (accessed on 4 November 2024)) [42].

### 2.5. Transwell Assay

Cells were seeded at a density of 50,000 cells/well (150,000 cells/cm^2^) into the upper chamber (insert) of the 96-well transwell plates (pore size 8 μm, polycarbonate membrane, Corning, Glendale, AZ, USA). The cells were stimulated to migrate through the membrane by medium supplemented with 5% of the chemoattractant fetal bovine serum (FBS) (PAA Laboratories, Pasching, Austria) in the lower chamber (reservoir). For testing of migration inhibition upon compound treatment, compounds were used at a concentration of BMC_10_ (V) of prior cMINC testing. Three technical replicates per condition were used. As a control for “unstimulated” migration, medium without any chemoattractant was tested. As an endpoint specific control for “inhibited” migration, CytoD with a concentration of 200 nM was used. After 6 h incubation time, the medium was aspirated, and reservoirs were washed with DPBS. Afterwards, reservoirs were filled with EDTA dissociation solution (DPBS supplemented with 0.1% 0.5 M EDTA UltraPure pH 8.0 (Thermo Fisher Scientific, Waltham, MA, USA), and 0.18% NaCl) containing 0.25 μL/mL calcein-AM and incubated for 30 min at 37 °C. The plates were then centrifuged at 300× *g* for 5 min (1200 rpm, Heraeus Multifuge 1 S-R, ThermoScientific) to remove migrated cells from the membrane. The tray with 96 wells of permeable inserts was removed, and the receiver plate was measured in the spectrophotometer (TECAN, Männedorf, Switzerland) with an emission wavelength of 520 nm. After subtraction of the blank values, the number of migrated cells was normalized to that of cells stimulated with FBS.

### 2.6. Seahorse Assessment of Mitochondrial Functional Parameters

The Seahorse bioanalyzer simultaneously measures cellular oxygen consumption rates (OCR) and extracellular acidification rates (ECAR). Therefore, NCCs were seeded at least two days prior to the assays at a density of 80,000 cells/well on Seahorse XF96 V3 PS Cell Culture Microplates (Agilent, Santa Clara, CA, USA) in either glucose medium or galactose medium. After 24 h, the medium was exchanged. For the real-time ATP production rate and mitochondrial stress assay, the cartridge was hydrated at 0% CO_2_ and 37 °C, and the seahorse was turned on to stabilize the temperature one day prior. The medium was changed to Seahorse XF DMEM Medium (Agilent, Santa Clara, CA, USA) supplemented with 2 mM L-glutamine, 1 mM pyruvate and 18 mM glucose or galactose 1 h before the start of the assays. Directly before the start of the assay, the medium was exchanged again. The sensor cartridge was loaded with the test compounds anchored to the BMC_10_ (V) of the cMINC screen and the recommended concentrations of calibration compounds for a mitochondrial stress test. These were 5 μM oligomycin, 15 μM carbonyl cyanide p-trifluoro methoxyphenylhydrazone (FCCP) and 5 μM rotenone plus 5 μM antimycin A. Additionally, 1 μg/mL H-33342 was added to the rotenone and antimycin A injection to perform afterwards a cell count (Cellomics, Waltham, MA, USA), to which the data were normalized. Six technical replicates were used for control conditions, and three technical replicates were used for other samples. The software Seahorse Analytics (Agilent, Santa Clara, CA, USA) was used for the evaluation (https://seahorseanalytics.agilent.com (accessed on 30 October 2024)).

### 2.7. Intracellular ATP Measurement

Cells were seeded at a density of 20,000 cells/well. The NCCs were treated with the BMC_10_ (V) of the cMINC screen for one hour, six hours or 24 h. Then, a CellTiterGlo-Triton mix was prepared. Therefore, a 0.5% Triton solution in PBS was prepared, and the commercial reagent mix CellTiterGlo 2.0 (Promgea, Madison, WI, USA) was added in a 1:1 ratio. An amount of 50 µL/well of the mix was added to 100 µL of medium. The plate was shaken for two minutes, and 100 µL of the cell mixture was transferred to a white measurement plate. For the blank value, only medium with the mix was measured. Luminescence data of samples were normalized to DMSO solvent controls, and the ATP data are given in % relative to “untreated cells”.

### 2.8. Glucose–Galactose Modified Experiments

To perform the experiments under mitochondria-dependent conditions [43], the cells were handled exactly in the same way, but the medium was adapted. The N2-S medium was adjusted, so that no glucose was present but galactose. The medium consisted of SILAC^TM^ Advanced DMEM/-F12 FLEX (Gibco/Fisher Scientific, Hampton, NH, USA), 15 μg/mL insulin, 400 μM GlutaMAX, 0.7 mM L-Arginine, 0.5 mM L-Lysine, 21.5 µM Phenol Red, 20 nM progesterone, 100 μM putrescine and 30 nM selenium, 92.5 μg/mL apotransferrin, 4.7 mg/mL galactose (18 mM). The cytokines 20 ng/mL EGF and 20 ng/mL FGF2 were always freshly added to each run.

### 2.9. PBK Modeling

A physiology-based kinetic (PBK) modeling was performed for picoxystrobin in the Simcyp Simulator V22 (Certara, UK) using a previously published approach [44]. The PBK model was parametrized using picoxystrobins’ physicochemical properties (molecular weight = 367.3, LogP = 3.68, polar surface area = 57.6, charge characterization = neutral). The in vitro measured values for the fraction unbound in blood plasma (=0.0127) and the blood to plasma ratio (=0.71) were used in parametrizing the simulations (measured by Cyprotex as part of EU Toxrisk). The steady-state volume of distribution (Vss) (=3.32 L/kg) and plasma to tissue partition coefficients (in both mother and fetus) were predicted by the method of Rodgers and Rowland [45]. Clearance was predicted by in vitro to in vivo extrapolation using in vitro Clint data generated in human hepatocytes. Binding to the hepatocytes in vitro was predicted as described by Kilford and colleagues [46]. Populations of 100 virtual pregnant subjects (aged between 18 and 45, at gestational week 20) were generated and used in the PBK simulations. The permeability in the intestine for picoxystrobin was predicted using the approach outlined originally by Sugano [47] and implemented within the Simcyp simulator as described earlier [48]. Partitioning of the compound into bile salt micelles and the solubility enhancement that this confers was modelled using the approach outlined by Sugano [49]. The details of the pregnancy population within Simcyp and performance verification using the pregnancy population have been described in the literature previously [50,51,52,53]. Similarly, the physiology data and approach to model the exposure to fetal tissues have been described in the literature [54,55,56,57]. The placental permeability of the compound was predicted using the polar surface area (PSA) and hydrogen bond donors (HBDs) of picoxystrobin using the approach described by Abduljalil and colleagues [58]. Picoxystrobin (dosed orally at 0.09 mg/kg of body weight per day) was used as the input dose for the PBK models.

### 2.10. Biokinetics Modeling

The modeling of the in vitro biokinetics of picoxystrobin was performed using the virtual in vitro distribution (VIVD) model by Fisher and colleagues [59] implemented in the SIVA toolkit (Certara Predictive Technologies). The VIVD model predicts the partitioning of a compound within an in vitro system at equilibrium into the various in vitro compartments (air, plastic, unbound in the medium, intracellular water, mitochondria, and lysosomes). The extent of compound binding to the medium depends on the compound’s physicochemical properties and the presence of lipids and/or proteins within the medium. To model picoxystrobins partitioning within an in vitro system at equilibrium, the following parameters were used: system temperature of 37° C, plastic container with a well volume of 360 µL, and the volume and pH of the medium as Vol = 125 µL and pH = 7.4. For NCCs, a membrane potential of −70 mV and a cell diameter of 12 µM (single cell volume = 0.45 pl were assumed) were used. The lipid composition of NCCs was assumed to be the default in SIVA (as in HepG2 cells). The volumetric fractions within the cells were 1% of lysosomes, 10% of mitochondria and 58.6% of intracellular water. The concentrations of lipids and proteins within the culture medium were parameterized for linoleic acid with 0.042 mg/L and negligible quantities of proteins. The physicochemical properties of picoxystrobin were modelled as follows: 3.6 LogP (US EPA (2012): Estimation Programs Interface Suite™ for Microsoft^®^ Windows, v 4.1, United States Environmental Protection Agency, Washington, DC, USA); 6 × 10^−4^ (Pa*m^3^*mol⁻^1^) Henry’s law constant at 25 °C; and a neutral compound. The model assumptions were that within cells, lipid binding is assumed to dominate compared to binding to proteins, which, in general, is considered to be a low-affinity binding; if it happens at high affinity, the concentration of the target is assumed to be low. Lipid concentration was assumed to be uniform across the cell compartments. Membrane potential differences were assumed to affect concentrations of charged compounds on both sides of the membrane.

## 3. Results and Discussion

### 3.1. Multi-Tiered Screening of a Compound Library in the cMINC Assay

**Figure 1:**
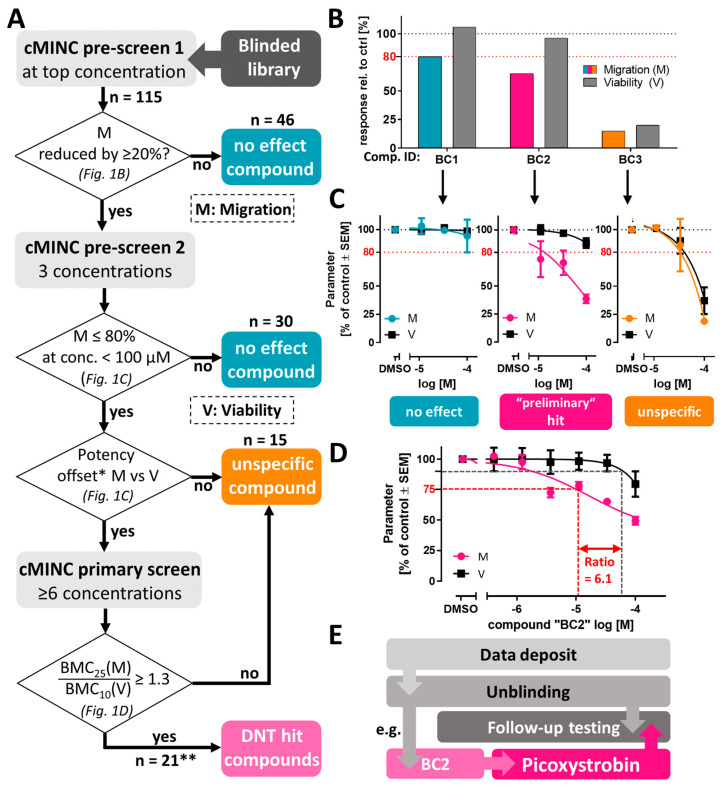
**Outline of the screen process and follow-up studies.** (**A**) A tiered testing strategy was applied to identify compounds that inhibit neural crest migration in the cMINC assay. The decision boxes indicate subfigures with exemplary details. (**B**) Exemplification of data resulting from cMINC pre-screen 1 on “blinded compounds” BC1, BC2 and BC3 (blinded at this stage, only compound IDs given). All shown compounds advanced to the next tier. Data for pre-screen 1 of selected compounds are given in Figures S2 and S3 (1N, 4n). (**C**) Exemplification of data resulting from cMINC pre-screen 2 for compounds shown in B. At this stage, 3 concentrations were tested, and compounds were classified based on the rules shown in A. Data for pre-screen 2 of selected compounds are given in Figure S4 (≥2N, 3n). (**D**) Full concentration–response curve for compound BC2 obtained in the primary screen. A ratio of BMC_25_ (M)/BMC_10_ (V) was calculated, and resulted in a hit call (≥3N, 3n). (**E**) After testing completion of all tiers, data were deposited at the NIEHS database. Subsequently, compounds were unblinded (e.g., BC2 was picoxystrobin). The hits were followed up in an orthogonal assay. * an offset of BMC_20_ (M) vs. BMC_20_ (V) of 2 was considered as an alert; ** 21 compounds were DNT hit calls. Four additional compounds were categorized as “borderline compounds”. BC: blinded compound.

A tiered testing strategy was applied to test a library of 115 blinded compounds in the cMINC assay (=UKN2). The screen comprised three stages: Pre-screen 1, pre-screen 2 and primary screen (Figure 1A). As with any tiered approach, there are trade-offs between resource efficiency and comprehensive initial testing. By prioritizing a streamlined and resource-saving strategy, we accepted potential limitations during the pre-screening phase. Data on compounds from pre-screening stages should be judged with caution, as they carry a risk of being false positives (FPs) or false negatives (FNs).

In the first tier (pre-screen 1), compounds were tested at the highest concentration only (in general 100 µM). For instance, the compounds with the ID “BC1”, “BC2” and “BC3” reached the threshold of reduced migration and/or viability. They were therefore followed up in pre-screen 2 (Figure 1B). They reduced the migration (M) readout by <20% (Figures S2 and S3). Altogether, 46 compounds were classified and excluded as “no effect”. One limitation of this pre-screen phase is that it relied on a single biological replicate. In future screens, a more sensitive threshold, such as BMR 85%, might be considered to improve sensitivity at this stage.

In the second tier (pre-screen 2), at least three concentrations were tested for the remaining 69 compounds. Thirty-nine compounds showed some effect. Fifteen of these compounds were labelled as “unspecific” toxicants, because their effect on migration was not more potent than that on viability (V). Compounds, which showed a potency offset between the migration and viability readouts, were classified as “preliminary hit” compounds (Figure 1C and Figure S4).

**Figure 2:**
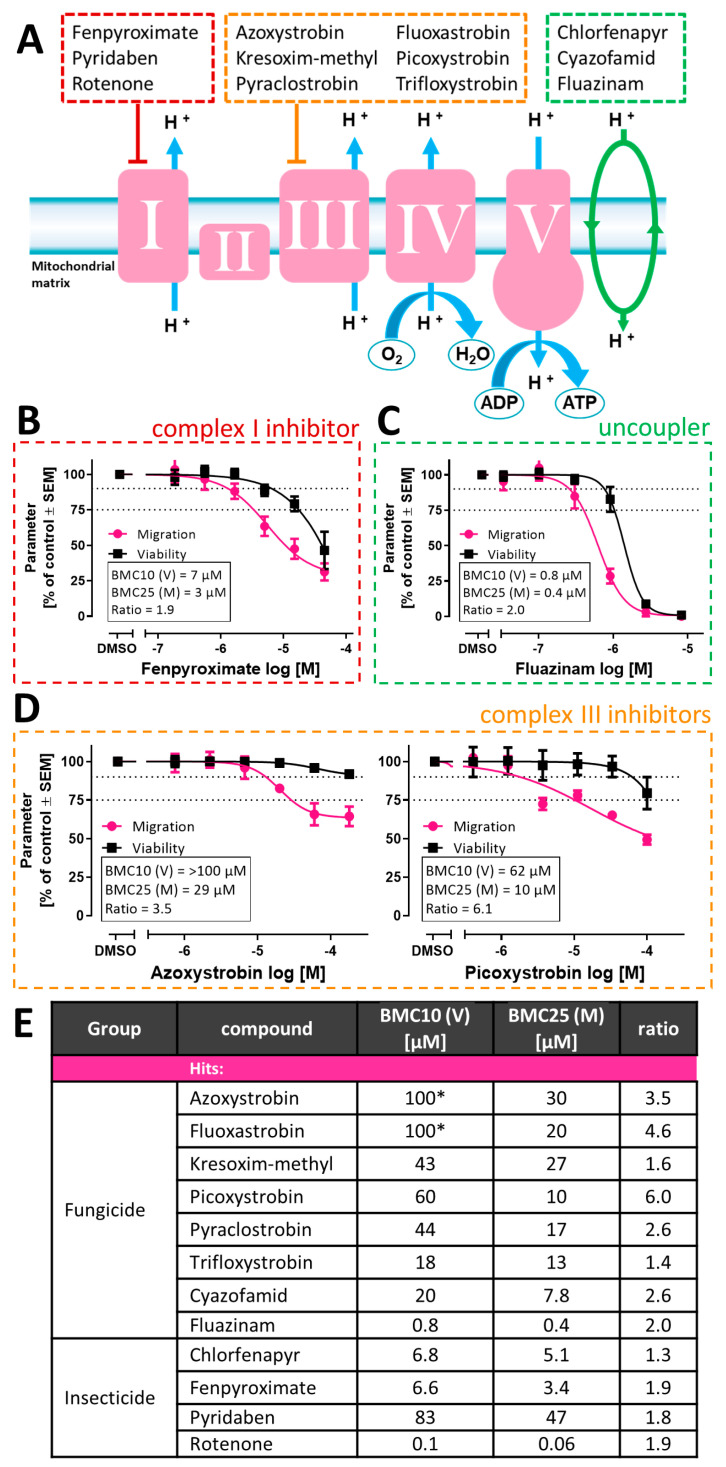
**Synopsis of screen data on mitochondria-related hits.** In total, 115 compounds were screened in the cMINC assay. After completion of the primary screen, i.e., the last tier of testing, 21 compounds were classified as hits. According to the published literature, 12 out of 21 specific hits from the cMINC screen targeted mitochondrial respiration. (**A**) Complexes (roman numbers) of the electron transfer chain are shown. The green ellipse symbolizes the effect of uncouplers. The assumed targets of 12 screen hits are indicated. (**B**) Concentration–response curve of fenpyroximate, an example of a complex I (cI) inhibitor. (**C**) Concentration–response curve of fluazinam, an example of an uncoupler. (**D**) Concentration–response curves of azoxystrobin and picoxystrobin, two examples of complex III (cIII) inhibitors. Data of other mitochondrial inhibitors are given in Figure S5. All data are from ≥3 biological replicates. The data in the insert boxes are derived from curve fitting of the data. (**E**) Tabular overview of the 12 specific mitochondrial hit compounds and their respective BMC_10_ (V) and BMC_25_ (M). BMC_25_ (M) was considered as the relevant threshold concentration for migration impairment. BMC_10_ (V) was assumed to be the highest non-cytotoxic concentration. It was used as a reference point for follow-up testing in an orthogonal assay. *: no effect could be observed even at the highest tested concentration (HTC). To calculate the ratio, the HTC is used.

In the last tier (primary screen), a full concentration response (at least six data points) was obtained for the 24 preliminary hit compounds. The highest tested concentration (HTC) was adjusted in cases where a high cytotoxicity was already observed during previous screening stages. A previously established prediction model [6,8] was applied to the obtained data to identify the 21 positive calls (hits) of this screen. The data for compound “BC2” with a migration/cytotoxicity ratio of 6.1 exemplify the prediction model (requiring a ratio ≥ 1.3) (Figure 1D). After unblinding, compounds were investigated for available information on their most likely mode of action (MoA) (Figure 2). The exemplary hit compound “BC2” was revealed as the fungicide picoxystrobin (Figure 1E and Figure S5). All further work concentrated on 12 selected hit compounds, as they belonged to a common mechanistic group. Other data will be deposited in a public database and described in other contexts.

### 3.2. Focus of Hit Follow-Up Activities on Mitochondrial Inhibitors

The MoA classification revealed that 12 of the 21 hits impair mitochondrial respiration (Figure 2A). Three hit compounds are known to inhibit complex I in the mitochondrial respiratory chain [60] (Figure 2A,B and Figure S5A). They comprised the pesticides fenpyroximate, pyridaben and rotenone. Three further pesticides are known as respiratory chain uncouplers (Figure 2A,C and Figure S5C): fluazinam, chlorfenapyr and cyazofamid [61]. The largest group (6-hit compounds) comprised complex III inhibitors (Figure 2A,D and Figure S5B) [61,62]. All compounds which inhibited complex III and were found to be specific in the cMINC assay belonged to the group of the strobilurin fungicides (Figure 2D). Picoxystrobin and fluoxastrobin showed the highest migration/cytotoxicity ratio of all screen compounds (Figure 2). The strobilurins azoxystrobin and fluoxastrobin were the only two compounds which did not reduce viability at all in the tested concentration range.

### 3.3. Effect of Mitotoxicants on ATP Levels and Mitochondrial Respiration

**Figure 3:**
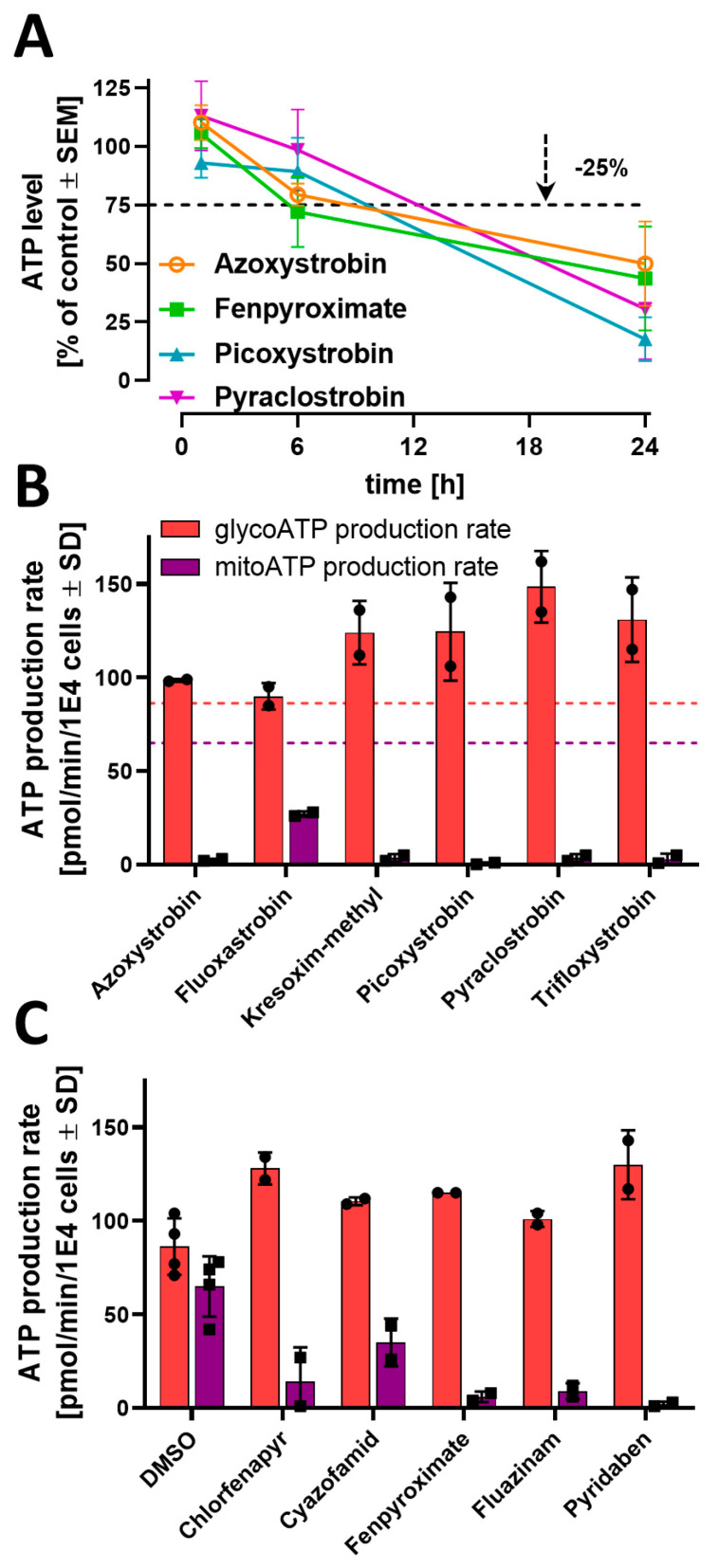
**Effect of mitochondrial toxicants on neural crest cell ATP levels and production.** (**A**) Effect of four mitochondrial toxicants on NCC ATP levels. ATP levels were measured at 1 h, 6 h and 24 h after addition to NCC cultures. A complete data set on other compounds is displayed in Figure S6. Data are expressed as means ± SEM from three independent biological replicates and are shown relative to the solvent control. (**B**,**C**) The effects of toxicants on ATP production rates are shown. Cells were treated with single concentrations corresponding to the BMC_10_ (V) of the cMINC screening (see Figure 2). Data on oxygen consumption rates under different metabolic conditions were used to calculate “glycoATP” as measure of the glycolytic ATP production rate and “mitoATP” as measure of mitochondrial ATP production rate. Dotted lines in (**B**) indicate the ATP production rate of cells exposed to solvent (0.1% DMSO). Data are expressed as means ± SD from two independent biological experiments.

We were interested to find out to which extent selected strobilurins affected NCC ATP levels. The cells were treated with the highest tolerated concentration, which was the BMC_10_ (V) of the cMINC assay. ATP levels were observed over time after exposure to azoxystrobin, picoxystrobin and pyraclostrobin (Figure 3A). As a reference compound, we included the well-known and potent complex I inhibitor fenpyroximate. ATP levels did not decrease after 60 min. A decrease in ATP levels by >25% was observed after 6 h of exposure to fenpyroximate. The strobilurin-treated cells still had ATP levels of >75% of control. Even after 24 h, all cell cultures had residual ATP levels of 25–50%, which is sufficient for all functions and survival [63,64,65]. Similar observations were made for the remaining strobilurins (trifloxystrobin, kresoxim-methyl, fluoxastrobin) (Figure S6A), the complex I inhibitors pyridaben and rotenone (Figure S6B), and the uncoupler cyazofamid (Figure S6C). Only the strong uncouplers fluazinam and chlorfenapyr caused a rapid decline of ATP by >25% after 1 h and by >90% after 24 h (Figure S6C).

The compounds were all used at concentrations known to compromise mitochondrial ATP generation [60,61,62]. In this light, the findings might appear surprising, but they are fully consistent with a large body of the literature: Cultured cells can compensate a loss of mitochondrial ATP generation by increased glycolysis [43]. To confirm this, we studied mitochondrial and glycolytic ATP production rates after exposure to mitotoxicants. For this, we measured NCC oxygen consumption in different metabolic states and used these primary data to calculate the two ATP production rates (glycoATP, mitoATP) [66,67,68]. Untreated NCCs (0.1% DMSO) had a glycolytic ATP production of about 85 pmol/min/10^4^ cells, and a mitochondrial rate of about 65 pmol/min/10^4^ cells. After direct (20 min) treatment with the strobilurins (Figure 3B) and the other mitochondrial inhibitors (Figure 3C), the mitochondrial ATP production dropped below the detection limit in nearly all cases. This confirms that all compounds were potent mitotoxicants in NCCs. In parallel, the glycolytic ATP production rate increased by about 50% (1.4-fold to 1.7-fold). These findings suggest that NCCs can maintain ATP levels sufficient for survival for long times, even when exposed to mitotoxicants. This is possible due to a high glycolytic contribution that is even increased in mitotoxicant-exposed cells. In summary, these findings suggest that a simple drop in ATP does not sufficiently explain the strongly inhibited migration capacity of NCCs exposed to mitotoxicants.

**Figure 4:**
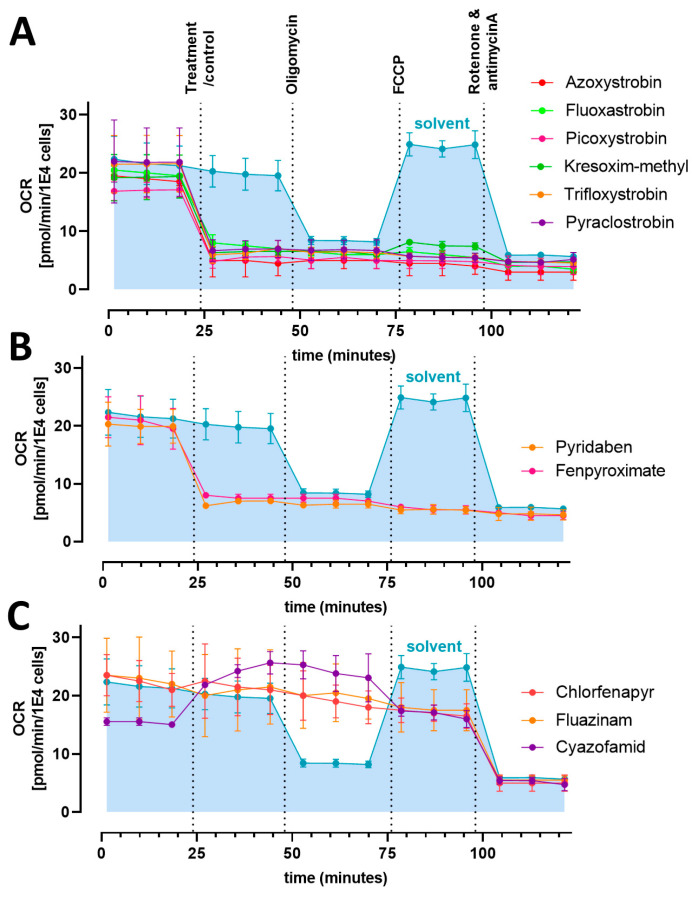
**Effect of mitochondrial toxicants on neural crest cell oxygen consumption.** The oxygen consumption rate (OCR) of NCCs was recorded. After baseline measurements for 20 min, cells were exposed to mitotoxicants at a concentration corresponding to the BMC_10_ (V) of the cMINC Screen (see Figure 2). Then, oligomycin, FCCP and rotenone/antimycin A were added sequentially, as indicated by dotted vertical lines. OCR data are normalized to the cell count and expressed as means ± SD from two independent biological experiments. (**A**) strobilurins/complex III inhibitors, (**B**) complex I inhibitors, (**C**) uncouplers.

Next, we investigated the effects of the mitotoxicants on the oxygen consumption rate (OCR) in NCCs. The OCR was studied in a seahorse analytical system after injection of the mitotoxicants (or control, 0.1% DMSO). The study protocol included a serial injection of the tool compounds oligomycin, FCCP and rotenone/antimycin A (Figure 4). In untreated conditions, injection of the control medium (0.1% DMSO) led to no change in OCR, i.e., the level of basal respiration was maintained. The subsequent injection of the complex V inhibitor oligomycin led to a decreased OCR due to the inhibited ATP-linked respiration. After an additional injection of the uncoupler FCCP, an increased OCR and the maximal respiration of the cells were observed. Finally, the injection of the complex I inhibitor rotenone together with the complex III inhibitor antimycin A led to a complete inhibition of the mitochondrial respiratory chain, and the OCR was decreased to a level, where only the non-mitochondrial oxygen consumption was recorded. In conclusion, untreated cells showed the expected oxygen consumption rates and a typical profile in this mitochondrial stress test protocol.

To investigate the effects of the mitotoxicants, the cells were treated with the highest tolerated concentration, i.e., the BMC_10_ (V) of the cMINC assay, after recording of the basal respiration. The injection of the complex III inhibitors led to a sudden drop of the OCR to the level of the non-mitochondrial oxygen consumption (Figure 4A). The injection of the three tool compounds did not alter the OCR. A similar effect was observed for the complex I inhibitors fenpyroximate and pyridaben (Figure 4B). Thus, all these compounds completely shut down the mitochondrial respiration.

The injection of the uncouplers fluazinam and chlorfenapyr caused no decrease in OCR (Figure 4C). Subsequent injection of oligomycin Click or tap here to enter text.also did not alter the OCR. Only the later injection of rotenone/antimycin A decreased the OCR to the level of non-mitochondrial oxygen consumption. This profile confirms an uncoupling effect of the compounds. Cyazofamid also showed a typical uncoupler profile in the NCC mitochondrial stress assay (Figure 4C). Upon the injection of cyazofamid, the OCR increased continuously and was unaffected by the oligomycin injection. Only the FCCP injection led to a small decrease in the OCR under the maximal respiration point. Similarly to the other uncouplers, the injection of rotenone/antimycin A showed a drop to the non-mitochondrial oxygen consumption level. In summary, the strobilurins quickly blocked mitochondrial respiration at a concentration that allowed a full or partial maintenance of cellular ATP levels over 6–24 h.

### 3.4. Conceptualization of a Candidate Adverse Outcome Pathway

**Figure 5:**
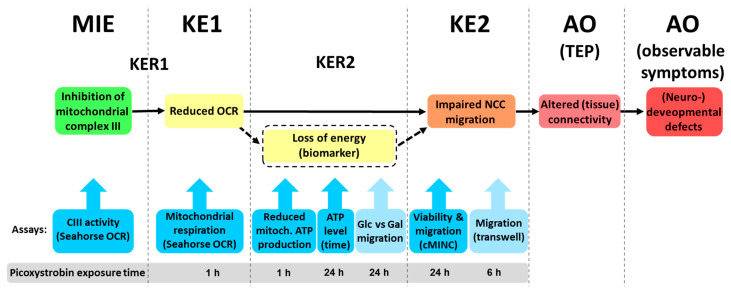
**Hypothetical AOP linking mitochondrial inhibition of neural crest cells to developmental toxicity.** A putative AOP was constructed. Below the AOP, we indicated potential assays to test KEs and their linkage. We picked the complex III inhibitor picoxystrobin as an exemplifying compound. Thus, the respective picoxystrobin assay exposure times used in this study are shown. MIE: molecular initiating event; KE: key event; KER: key event relationship; darker blue boxes indicate assays used to establish the AOP; lighter blue boxes indicate assays that can confirm the AOP; AO: adverse outcome; TEP: toxicity endophenotype; cIII: mitochondrial complex III; OCR: oxygen consumption rate; Glu vs. Gal: glucose vs. galactose medium conditions; biomarker: could also be a modifying factor of KER2, but needs more research.

To guide our understanding of how mitochondrial complex III inhibition in NCCs could result in (neuro)developmental defects, we collected literature information on potential key events (KEs) and their biological inter-relationships. We also searched for assays that would assess KEs and important modulatory factors. This information was used to suggest a putative adverse outcome pathway (AOP) (Figure 5), which is described here following the rationale that led to its assembly: We considered “impaired NCC migration”, i.e., the primary outcome of the phenotypic screen as KE2 of this AOP. NCC migration has been described extensively as a fundamental neurodevelopmental process [8,13] and therefore qualifies as AOP KE. The fit with basic principles of AOP construction is given, as this KE is conveniently measured by the cMINC (UKN2) assay, and compounds active in this assay may lead to an altered connectivity of the brain, i.e., a DNT endophenotype.

We linked KE2 directly to the adverse outcome (AO), as we did not have sufficient information on another essential and measurable event further downstream. Notably, we suggest defining the AO based on an “altered connectivity state”. We suggest not referring to a specific behavioral outcome (exophenotype), as it cannot be defined from the effects of toxicants in NAM. We feel that our approach is in line with a toxicological tradition that uses “internal measures”, such as histology or nerve conductance velocity, e.g., to define nerve injury.

Following the strategy used earlier for AOP: 3 [69], the binding and inhibition of complex III in NCCs was defined as the molecular initiating event (MIE). This accounts for the vast amount of data on mitochondrial inhibition by strobilurins [60,61]. We used a molecular docking simulation to provide further evidence for binding of picoxystrobin to mitochondrial complex III (Figure S8). This docking of picoxystrobin in the binding pockets of Cyt b was also already shown for, e.g., *Colletotrichum truncatum* [70]. In addition, the inhibition of cIII was tested in NCCs. Our data confirmed the inhibition of complex III of the mitochondrial respiratory chain by picoxystrobin (Figure S9).

As linking KEs between the MIE and KE2, we suggest a decrease in mitochondrial respiration as KE1. This KE is assessed by the measurement of the oxygen consumption rates. Strobilurins indeed blocked this KE (Figure 4). We initially considered “cellular energy/ATP-depletion” as potential additional KE, but decided against it as the essentiality of this event is currently not sufficiently proven. For instance, ATP levels of NCCs were maintained at relatively high levels after strobilurin exposure (Figure 3A). We suggest that energy depletion may be a biomarker for KE1 under some (but not all) conditions.

Key event relationship 2 (KER2) describes how reduced respiration leads to impaired migration. ATP depletion is currently best described as a modifying factor of KER2, in that it accelerates the transition or increases the likelihood for it to happen. To better describe and understand this transition, we used different assays and also took account of the exposure time to strobilurins (Figure 3). We considered additional assays to further explore KER2 and its link to KE2:

First, we propose here to measure migration within a more sharply defined time period after strobilurin exposure. An experimental approach to this is the transwell migration assay, performed below.

Second, we suggest verifying that inhibited respiration (metabolic activity) directly leads to KE2 (impaired migration). For this, we suggest running the cMINC in the presence (Glu = glucose) or absence (Gal = galactose) of mitochondrial substrates. An increased sensitivity of NCC migration to picoxystrobin under Gal conditions would increase the confidence level of our postulated AOP. Data on such experiments were generated in the next steps.

### 3.5. Hit Confirmation in an Orthogonal Migration Assay

**Figure 6:**
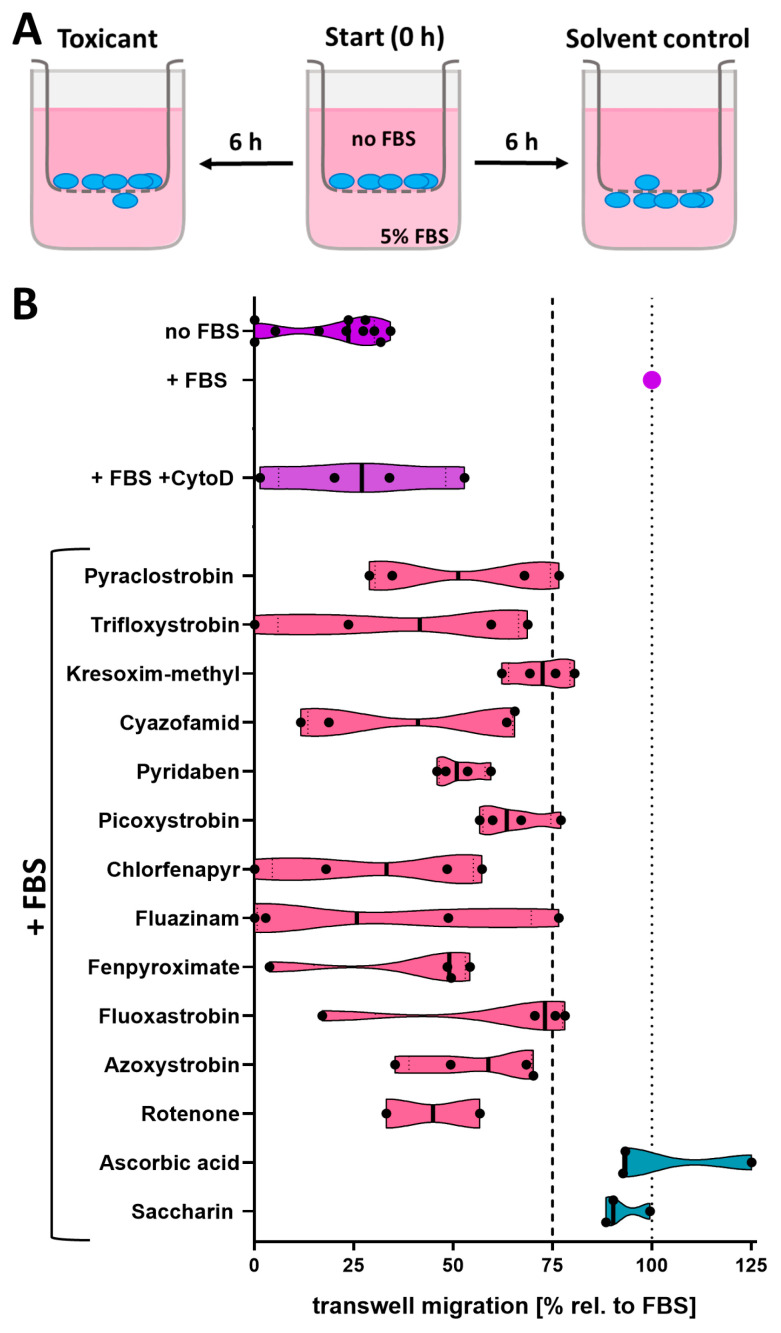
**Setup and performance of the neural crest transwell migration assay.** (**A**) Schematic illustration of the transwell migration assay. In the beginning, the NCCs are plated into the transwell inserts. The difference in FBS concentration between the upper and lower compartment stimulates NCCs to migrate through the membrane pores. Toxicants were applied in both compartments. After 6 h, the number of cells that reached the downward surface of the membrane was quantified. (**B**) Results of compound testing in the transwell assay: For calibration of the assay, cytochalasin D (CytoD) was used as positive control. Omission of FBS (no FBS) was used as second control for “inhibited” migration (shown in purple); pink: hit compounds of cMINC screen known to affect mitochondrial respiration; blue: negative controls of cMINC screen. All compounds were tested at a single concentration corresponding to the BMC_10_ (V) from the cMINC screen (see Figure 2E). Transwell migration is measured as the ratio of “migrated cells in the presence of toxicants to the number of migrated cells in the absence of toxicant”. The dotted line at 75% indicates the threshold for classification of compounds as specific migration inhibitors in the transwell assay. The black line in the violin plots represents the median. The black dots represent data from individual experiments. Data are from ≥2 independent biological experiments. FBS: fetal bovine serum.

To confirm the migration inhibiting effect of the mitochondrial primary screen hits in the cMINC assay, an orthogonal migration assay was performed (Figure 6). The adapted Boyden chamber assay, the so-called transwell assay (Figure 6A), was used. This test was already earlier established for NCCs [7,15]. The assay principle differs from the cMINC assay in that NCCs migrate along a chemo-attractive gradient (FBS as chemoattractant), and the migration time (and accordingly compound exposure) is shorter (6 h vs. 24 h).

The standard positive control for the assay, cytochalasin D (CytoD), led to 75% reduced migration of the NCCs. The test concentration of all mitochondrial toxicants was the BMC_10_ (V) of the cMINC assay, i.e., the maximal non-cytotoxic concentration. All test compounds reduced the migration by at least 25% compared to the control. When NCCs were treated with the negative controls ascorbic acid and saccharin (at high concentrations, 100 µM), no inhibited migration was observed. In summary, we fully confirmed the migration-inhibiting effect of the mitochondrial hits in an orthogonal assay.

### 3.6. Extended Testing and Data Generation Approaches to Provide Input for Risk Assessment

We chose one of the strobilurins (picoxystrobin) for an exemplary case study. A new stock of picoxystrobin was prepared from a different substance lot, to start this study from a “new” solid basis. Picoxystrobin was retested by two different operators (Figure S7A). The original data on inhibition of NCC migration were essentially confirmed, and data were operator-independent.

**Figure 7:**
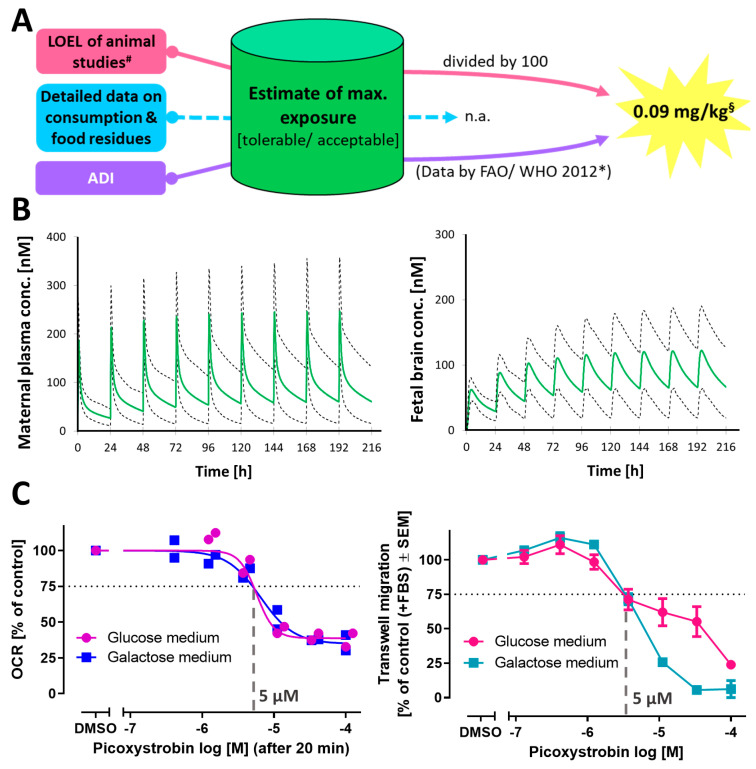
**Comparison of internal exposure estimates and primary effect potency.** (**A**) Schematic illustration of approaches to arrive at an estimate of a maximal (tolerable) exposure level of picoxystrobin. For picoxystrobin, no current data on consumption and food residues are available from EFSA. In an alternative approach, the lowest observed effect level (LOEL) of animal studies was used (9 mg/kg/day). By assuming a standard safety factor of 100, we estimated a human daily threshold dose of 0.09 mg/kg. In a second approach, we used the acceptable daily intakes (ADIs) suggested in a 2012 report of a joint meeting of FAO/WHO (REF: https://www.fao.org/3/i3111e/i3111e.pdf (accessed on 15 June 2024)). Both scenarios lead to the same upper exposure limit for picoxystrobin of 0.09 mg/kg (per day). (**B**) A physiologically based kinetic (PBK) model was established for picoxystrobin. The model was parametrized to reflect a population of pregnant subjects in gestational week 20, and their foetus, with a daily intake of 0.09 mg/kg (see (**A**)), was modelled. The predicted concentrations of picoxystrobin are shown. Data (green lines) are population averages of pregnant subjects (n = 100), aged between 18 and 45. The dashed lines indicate the 5th and 95th percentiles of the population. (**C**) The left graph shows the concentration–response curve for the oxygen consumption rate (OCR) of NCCs directly (20 min offset) after the picoxystrobin injection. Measurements were performed in glucose or galactose medium. Data are shown for two independent experiments. Each data point shown is the average of three technical replicates. The right graph shows the concentration–response curve of picoxystrobin in the transwell assay. The assay was performed either in glucose or galactose medium. Data are expressed as means ± SEM from three independent biological experiments. ^#^ LOEL of animal study is based on a 90-day dog study (REF: https://www.fao.org/3/i3111e/i3111e.pdf (accessed on 15 June 2024)). *^,§^ There is also an old (no longer valid) value by EFSA of 0.043 mg/kg/day from the year 2004 [71].

An estimate of the maximal external exposure was obtained as the next step of the study. We used two different approaches, based on (i) setting the acceptable daily intake as the upper limit and (ii) referring to the no-observed-effect level (NOEL) in an animal study (detailed in Figure 7A). We concluded from both approaches that a plausible upper daily tolerated threshold is 0.09 mg/kg. This value was used in a physiologically based kinetic (PBK) model to obtain concentration time data for different tissues in mothers and their fetus. Assuming a repeated daily exposure, the maximal maternal plasma concentrations were predicted to be in the 200–300 nM range. The maximal steady-state fetal brain concentrations were predicted to be in the 100 nM range (5th and 95th percentiles for exposure in the population of 100 subjects were 60–190 nM) (Figure 7B). These data were used later for comparisons of a potential internal exposure with points of departure (POD) for toxicity. Next, we used some advanced assays to obtain a better understanding of the potency range of picoxystrobin when used in different experimental setups. This information was intended to allow us to define a sufficiently sensitive POD for risk assessment. To start with, we tested whether it made a difference to work with NCCs cultured in the presence (Glu) or absence (Gal) of glycolytic substrates [43]. Under both conditions, acute exposure to picoxystrobin blocked respiration at about 5 µM. This finding was in line with our expectations, as direct target interaction (inhibition of c-III) and subsequent block of the electron transport chain (ETC) should be independent of the culture medium. Instead, the functional consequence (NCC migration) might have differed, as exposure to an ETC inhibitor in galactose medium leads to a more rapid depletion of cellular ATP than the same toxicant concentration in glucose medium [43]. We found that the culture conditions made no difference in the transwell migration assay. Exposure time in this assay was 6 h only. Migration was inhibited at concentrations ≥ 5 µM in both media. We conclude that the dependence on mitochondria for ATP production and differential ATP levels in cells do not affect the POD after acute exposure or in short-term (few hours) assays. This is consistent with our earlier observation (Figure 3A) that migration was affected in the cMINC assay also under conditions that affected ATP only to a minor extent.

**Figure 8:**
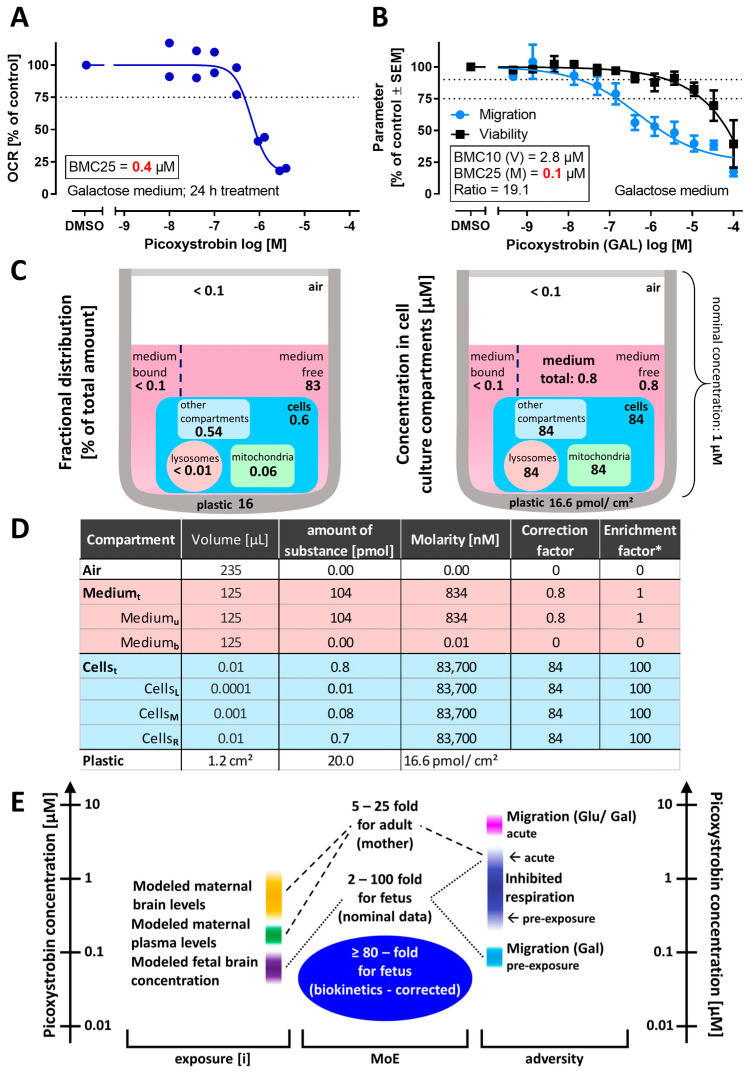
**Consideration of biokinetics for refined hazard (potency) estimates.** (**A**) Concentration–response curve of the oxygen consumption rate (OCR) in NCCs cultured in galactose medium after a 24 h treatment with picoxystrobin. The highest tested concentration was 2.8 µM (highest non-cytotoxic exposure). Data are shown for two independent experiments (as in Figure 7B). (**B**) The cMINC assay was performed as in Figure 1, but the NCCs were cultured in galactose medium. The insert box gives picoxystrobin potency data for migration (M) and cytotoxicity (V), and their ratio. Data are expressed as means ± SEM from seven independent experiments. (**C**) Schematic illustration of the distribution of picoxystrobin in a cell culture well according to the in silico biokinetics prediction model. Data for each compartment are given either as percentage (left) or as concentrations (right) for a nominal concentration of 1 µM. (**D**) Tabular overview of the distribution of picoxystrobin in the different compartments at a nominal concentration of 1 µM. Medium_t_: total medium; Medium_b_: bound in medium; Medium_u_: unbound in medium; Cells_t_: total amount in cells; Cells_M_: mitochondrial compartment; Cells_L_: lysosomal compartment; Cells_R_: “rest” of the cells. The correction factor indicates the change vs. the nominal concentration. * The enrichment factor is defined as the distribution ratio of the compound in the compartments vs. the medium. (**E**) Synoptic overview of predicted and measured concentrations of picoxystrobin. Data on internal exposure in humans (left) are from the PBK model (Figure 7). Right: the concentration ranges at which picoxystrobin showed adverse effects in the experiments (e.g., migration inhibition in NCCs). The margins of exposure (MoE) for the mother and the fetus were estimated from these data by forming the ratios of hazard concentrations and exposure concentrations. For the fetal hazard concentrations, we considered (i) an upper limit, defined by the results of (acutely) inhibited respiration (see Figure 7C) and (ii) a lower limit defined by the results of inhibited migration in Gal medium (see (**B**)). For the hazard concentration in an adult, the inhibited respiration after 24 h exposure was used (see (**A**)). Exposure data used here were the modelled fetal brain concentration (100 nM range) and the maternal plasma concentration (200–300 nM range) (see Figure 7B). The fetal brain concentration was also used for the biokinetics-corrected MoE; here, the modelled concentration in the cells was used instead of the nominal concentration (see (**C**)). Exposure[i]: internal exposure measure in concentration (molarity) units. MoE: ratio of “minimally toxic concentration” and exposure[i].

We also tested some parameters important for model predictions of human threshold doses, e.g., the presence of human serum albumin (HSA). The medium was supplemented with 1% HSA. The addition of 1% HSA did not alter the effect of picoxystrobin on NCC migration (Figure S7B). We also reasoned that compound accumulation over time may play a role in picoxystrobin toxicity. In order to check this, NCCs were pre-treated for 24 h with picoxystrobin. When the OCR was measured under these conditions, the POD was lowered 10-fold (to 0.4 µM) (Figure 8A). We followed up on this by checking the functional consequences in the cMINC assay in galactose medium. The apparent potency was strongly increased, as migration was now inhibited in the 100 nM range (Figure 8B). This observation was similar for an adapted transwell assay (Figure S11A). NCCs were pre-treated with picoxystrobin for 18 h and then stimulated to migrate for 6 h. Here, migration was inhibited in the 600 nM range (Figure S11B). These data suggest that for a continuous exposure to picoxystrobin, or any other schedule that allows for a longer contact with target cells, nominal concentrations as low as 100 nM may lead to adverse effects.

In the context of the above findings, it was important to better understand how nominal concentrations related to cellular (or tissue) concentrations. To obtain such estimates, we used an in vitro cell distribution model [59]. The following compartments were considered: plastic, air, cells and medium. For modeling purposes, the cells were simplified and assumed to consist of three compartments: mitochondria, lysosomes, and the “rest” of the cells (=other compartments). Most of the picoxystrobin was predicted to be free (unbound) in the medium (83%) or sticking to the plastic of the plate (16%). Less than 1% entered the cells. However, when the volume of the cells was considered (0.45 µL/10^6^ cells), this meant a 100-fold accumulation (relative to medium), i.e., a nominal concentration of 1 µM produced values around 80 µM inside cells or mitochondria (Figure 8C,D). This knowledge is important for in vitro-to-in vivo extrapolations (IVIVE). For instance, it would seem a reasonable assumption that the intracellular concentration corresponds to the target tissue concentration in humans. This information can be used as a starting point for reverse PBK modeling of the corresponding dose. Alternatively, this information may be used to convert POD obtained as “nominal concentrations” into “cellular concentration POD” (ccPOD). We considered this correction an important step in risk assessment. Here, it would mean that a nominal POD of 100 nM would correspond to a ccPOD of 8–10 µM. Using this assumption for IVIVE would mean that adverse effects are observed at a tissue concentration of ≥8 µM.

This case study does not attempt any risk assessment as such. It was rather undertaken to explore which follow-up steps need to be taken once a compound was identified as a hit in a screening assay relevant to toxicity. The various types of approaches exemplified here may be used as inputs to risk assessment. One step in this method that we still missed was a transparent synopsis of the various categories of data. We chose to display relevant maximal internal exposures along a concentration scale (Figure 8E). This visualization suggests that maternal toxicity needs to be considered differently from fetal toxicity (NCCs are only found in fetuses, but other cells, possibly targeted by picoxystrobin are also found in mothers). We chose to visualize the PODs from different assays used in this study alongside the internal exposures. The large potency difference observed for picoxystrobin in various assay formats suggests that it is important to select the most relevant NAM and to provide a rationale for this selection (Figure 8E).

Finally, we compared PoDs and internal exposure levels by calculating a “margin of exposure” (MoE), i.e., a ratio of PoD vs. the internal exposure (exposure[i]). This comparison showed quickly that the minimal concentration at which migration (cMINC in Gal) is inhibited and the modelled concentration in the fetal brain are at a similar level. For the fetus, the MoE ranged from 2- to 100-fold. These values are based on a fetal brain concentration of 0.05 µM and on either the highest or lower PoD determined here. However, if the (VIVD-modelled) intracellular accumulation of picoxystrobin in vitro was taken into account, the lowest hazard concentration increased to 8 µM, resulting in an MoE greater than or equal to 80-fold. For the mother, the PoD was assumed to be 5 µM in medium (block of respiration). Compared with a plasma concentration of 200 nM or maximal brain concentrations up to 1 µM (Figure S10), we calculated a MoE of 5–25. The in vivo PBK and in vitro cell disposition modeling undertaken here was a simulation exercise using available in vitro and physicochemical data as model input. To be used in a formal risk assessment exercise, the uncertainty associated with the model outputs would also need to be considered. Nevertheless, this an important concept when trying to put the in vitro hazard data for picoxystrobin into the context of a risk assessment.

## 4. Conclusions

The integration of NAM into toxicological assessments is gaining momentum, but their acceptance still depends on calibration against reference data. For DNT, such reference data predominantly come from OECD TG426 [72] and TG443 [16] guideline studies. To date, OECD TG426 has been applied to approximately 200 compounds, with many results deemed outdated or inconclusive (report in preparation (2024) by K. Crofton). The newer TG443 guideline, incorporating a DNT cohort, has been used for only 45 substances out of more than 5000 high-production-volume chemicals (>1000 tons per year; ECHA and TSCA database). Notably, in Europe, only 25 marketed pesticides have undergone TG426 testing. This lack of data underscores the importance of driving forward NAM-based strategies.

Here, we exemplified a robust screening strategy centered around a NAM that is relevant to developmental and reproductive toxicity (DART), and that also contributes to the recently described test battery for DNT (DNT-IVB, Blum et al. [5]; OECD [73]). A major part of this study provides a template for hit follow-up. We intend to outline a bridge from screening approaches to risk assessment.

Our study addresses a fundamental question of the DNT-IVB [8]: how should a tiered follow-up strategy look once a compound is found as a hit in one of the NAMs of the test battery? We provide here a case study that exemplifies the follow-up assessment of screening hits and improves the interpretation of the screen data. After unblinding of screen data, a group of mechanistically related hits emerged: inhibitors of c-III of the mitochondrial respiratory chain. We investigated this mechanistic cue more closely, as it pointed towards a new AOP, not considered hitherto for neural crest cell toxicity.

While some of the hit characterization steps are specifically tailored to picoxystrobin, most of the approaches shown here have a broad applicability. For instance, the potential grouping of hits is one of the generally applicable first approaches for every screen. In our study, there was a high over-representation of mitochondrial toxicants as specific hits (n = 12), and six compounds were even chemically related (strobilurins). This finding already gives important directions for follow-up studies. Not every screen will result in such a favorable situation with a straightforward mechanistic hypothesis to explain most hits. However, many strategies have been developed to identify targets from phenotypic screens, e.g., using combined omics technologies and/or advanced bioinformatics approaches [74,75,76,77,78].

Here, we also highlighted approaches that may be taken by future studies. A roadmap for hit confirmation will be required [79]. A broad range of case studies appears necessary to optimize a general strategy, as outlined earlier in general strategy papers [80,81,82,83,84]. Following this objective, the intention of our study was not to come up with an actual risk assessment statement on, e.g., picoxystrobin. We rather exemplified general principles, and we indicated gaps that need to be closed for use of the approach in a real-life risk assessment.

While our follow-up experiments provided a deeper insight into the effects of picoxystrobin on the NCCs, the main purpose for this was not the mechanistic clarification as such. Instead, we undertook this effort to provide a better data basis to use NAM data for risk assessment.

One important learning was that the link of a screen hit to a putative adverse outcome pathway (AOP) largely facilitates the evaluation of its toxicological relevance [69]. As inhibition of the migration of NCCs is a key neurodevelopmental process (KNDP), it can be directly related to altered brain connectivity [8,13]. Therefore, the potential toxicological implications of the hits of this particular screen were relatively straightforward. Another learning was that exposure timing may play an important role in data interpretation. Detailed solutions on how to implement this for NAM-based risk assessment still need to be worked out.

A key step on the way from screen hits to risk assessment is the conversion of assay concentrations to estimated adverse doses [85,86]. We provided here an example on how exposure assessment may be integrated with kinetic modeling, including the assessment of intracellular drug concentrations. While we indicated and exemplified the building blocks of such a strategy, the exact definition and full quantification of all steps requires more case studies and more detailed work.

With an increasingly extensive and complex hit follow-up, a large set of data (with diverse data types) may be generated. We suggest here, to use and further develop approaches to make the essential information from this easily understandable and traceable. One exemplification here is the synoptical, graphical overview of hazard and exposure data, which could, in the future, be supplemented by defined measures of uncertainty.

Overall, we exemplify here how NAM-based high-throughput screening (including hit confirmation) may identify DNT alert compounds, add mechanistic understanding to these alert substances, and ultimately, may be used to support NAM-based risk assessment in the future.

## Data Availability

Additional raw data can be requested from the corresponding author.

## Supplementary Materials

The following supporting information can be downloaded at: https://www.mdpi.com/article/10.3390/cells13242057/s1. Figure S1: cMINC (UKN2) exposure scheme and imaging exemplification; Figure S2: Results of Pre-Screen 1; Figure S3: Pre-Screen 1 synopsis of results; Figure S4: Overview of results from Pre-Screen 2; Figure S5: Exemplary primary screen hits; Figure S6: ATP level after exposure to some mitochondrial toxicants; Figure S7: Confirmation testing of picoxystrobin using a new stock; Figure S8: Docking of picoxystrobin at complex III; Figure S9: Inhibition of electron transport chain complexes by picoxystrobin in neural crest cells; Figure S10: Modelling of maternal brain concentrations of picoxystrobin; Figure S11: Prolonged exposure of NCC in a modified transwell assay setup.

## References

[1] Zhao R., Trainor P.A. (2023). Epithelial to mesenchymal transition during mammalian neural crest cell delamination. Semin. Cell Dev. Biol..

[2] Le Douarin N.M. (2004). The avian embryo as a model to study the development of the neural crest: A long and still ongoing story. Mech. Dev..

[3] Szabó A., Mayor R. (2018). Mechanisms of Neural Crest Migration. Annu. Rev. Genet..

[4] Amiel J., Sproat-Emison E., Garcia-Barcelo M., Lantieri F., Burzynski G., Borrego S., Pelet A., Arnold S., Miao X., Griseri P. (2008). Hirschsprung disease, associated syndromes and genetics: A review. J. Med. Genet..

[5] Vega-Lopez G.A., Cerrizuela S., Tribulo C., Aybar M.J. (2018). Neurocristopathies: New insights 150 years after the neural crest discovery. Dev. Biol..

[6] Nyffeler J., Karreman C., Leisner H., Kim Y.J., Lee G., Waldmann T., Leist M. (2017). Design of a high-throughput human neural crest cell migration assay to indicate potential developmental toxicants. ALTEX.

[7] Nyffeler J., Dolde X., Krebs A., Pinto-Gil K., Pastor M., Behl M., Waldmann T., Leist M. (2017). Combination of multiple neural crest migration assays to identify environmental toxicants from a proof-of-concept chemical library. Arch. Toxicol..

[8] Blum J., Masjosthusmann S., Bartmann K., Bendt F., Dolde X., Donmez A., Forster N., Holzer A.K., Hubenthal U., Kessel H.E. (2023). Establishment of a human cell-based in vitro battery to assess developmental neurotoxicity hazard of chemicals. Chemosphere.

[9] Crofton K.M., Mundy W.R. (2021). External Scientific Report on the Interpretation of Data from the Developmental Neurotoxicity In Vitro Testing Assays for Use in Integrated Approaches for Testing and Assessment. EFSA Support. Publ..

[10] Aschner M., Ceccatelli S., Daneshian M., Fritsche E., Hasiwa N., Hartung T., Hogberg H.T., Leist M., Li A., Mundi W.R. (2017). Reference compounds for alternative test methods to indicate developmental neurotoxicity (DNT) potential of chemicals: Example lists and criteria for their selection and use. ALTEX.

[11] Grandjean P., Landrigan P.J. (2014). Neurobehavioural effects of developmental toxicity. Lancet Neurol..

[12] Grandjean P., Landrigan P.J. (2006). Developmental neurotoxicity of industrial chemicals. Lancet.

[13] Bal-Price A., Hogberg H.T., Crofton K.M., Daneshian M., FitzGerald R.E., Fritsche E., Heinonen T., Bennekou S.H., Klima S., Piersma A.H. (2018). Recommendation on test readiness criteria for new approach methods in toxicology: Exemplified for developmental neurotoxicity. ALTEX.

[14] Nyffeler J., Chovancova P., Dolde X., Holzer A.K., Purvanov V., Kindinger I., Kerins A., Higton D., Silvester S., van Vugt-Lussenburg B.M.A. (2018). A structure-activity relationship linking non-planar PCBs to functional deficits of neural crest cells: New roles for connexins. Arch. Toxicol..

[15] Dolde X., Karreman C., Wiechers M., Schildknecht S., Leist M. (2021). Profiling of Human Neural Crest Chemoattractant Activity as a Replacement of Fetal Bovine Serum for In Vitro Chemotaxis Assays. Int. J. Mol. Sci..

[16] OECD (2018). Test No. 443: Extended One-Generation Reproductive Toxicity Study. OECD Guidelines for the Testing of Chemicals, Section 4.

[17] Sachana M., Bal-Price A., Crofton K.M., Bennekou S.H., Shafer T.J., Behl M., Terron A. (2019). International Regulatory and Scientific Effort for Improved Developmental Neurotoxicity Testing. Toxicol. Sci..

[18] Carstens K.E., Carpenter A.F., Martin M.M., Harrill J.A., Shafer T.J., Friedman K.P. (2022). Integrating Data From In Vitro New Approach Methodologies for Developmental Neurotoxicity. Toxicol. Sci..

[19] Sachana M., Shafer T.J., Terron A. (2021). Toward a Better Testing Paradigm for Developmental Neurotoxicity: OECD Efforts and Regulatory Considerations. Biology.

[20] Behl M., Ryan K., Hsieh J.H., Parham F., Shapiro A.J., Collins B.J., Sipes N.S., Birnbaum L.S., Bucher J.R., Foster P.M.D. (2019). Screening for Developmental Neurotoxicity at the National Toxicology Program: The Future Is Here. Toxicol. Sci..

[21] Leist M., Hasiwa N., Rovida C., Daneshian M., Basketter D., Kimber I., Clewell H., Gocht T., Goldberg A., Busquet F. (2014). Consensus report on the future of animal-free systemic toxicity testing. ALTEX.

[22] Delp J., Gutbier S., Klima S., Hoelting L., Pinto-Gil K., Hsieh J.H., Aichem M., Klein K., Schreiber F., Tice R.R. (2018). A high-throughput approach to identify specific neurotoxicants/ developmental toxicants in human neuronal cell function assays. ALTEX.

[23] Shafer T.J., Brown J.P., Lynch B., Davila-Montero S., Wallace K., Friedman K.P. (2019). Evaluation of Chemical Effects on Network Formation in Cortical Neurons Grown on Microelectrode Arrays. Toxicol. Sci..

[24] Lee J., Schlichting R., Konig M., Scholz S., Krauss M., Escher B.I. (2022). Monitoring Mixture Effects of Neurotoxicants in Surface Water and Wastewater Treatment Plant Effluents with Neurite Outgrowth Inhibition in SH-SY5Y Cells. ACS Environ. Au.

[25] Renner H., Grabos M., Becker K.J., Kagermeier T.E., Wu J., Otto M., Peischard S., Zeuschner D., TsyTsyura Y., Disse P. (2020). A fully automated high-throughput workflow for 3D-based chemical screening in human midbrain organoids. eLife.

[26] Jarema K.A., Hunter D.L., Hill B.N., Olin J.K., Britton K.N., Waalkes M.R., Padilla S. (2022). Developmental Neurotoxicity and Behavioral Screening in Larval Zebrafish with a Comparison to Other Published Results. Toxics.

[27] Thunga P., Truong L., Tanguay R.L., Reif D.M. (2021). Concurrent Evaluation of Mortality and Behavioral Responses: A Fast and Efficient Testing Approach for High-Throughput Chemical Hazard Identification. Front. Toxicol..

[28] Zurlinden T.J., Saili K.S., Rush N., Kothiya P., Judson R.S., Houck K.A., Hunter E.S., Baker N.C., Palmer J.A., Thomas R.S. (2020). Profiling the ToxCast Library With a Pluripotent Human (H9) Stem Cell Line-Based Biomarker Assay for Developmental Toxicity. Toxicol. Sci..

[29] Zimmer B., Pallocca G., Dreser N., Foerster S., Waldmann T., Westerhout J., Julien S., Krause K.H., van Thriel C., Hengstler J.G. (2014). Profiling of drugs and environmental chemicals for functional impairment of neural crest migration in a novel stem cell-based test battery. Arch. Toxicol..

[30] Knudsen T.B., Houck K.A., Sipes N.S., Singh A.V., Judson R.S., Martin M.T., Weissman A., Kleinstreuer N.C., Mortensen H.M., Reif D.M. (2011). Activity profiles of 309 ToxCast chemicals evaluated across 292 biochemical targets. Toxicology.

[31] Judson R.S., Houck K.A., Kavlock R.J., Knudsen T.B., Martin M.T., Mortensen H.M., Reif D.M., Rotroff D.M., Shah I., Richard A.M. (2010). In vitro screening of environmental chemicals for targeted testing prioritization: The ToxCast project. Environ. Health Perspect..

[32] Wetmore B.A., Wambaugh J.F., Ferguson S.S., Sochaski M.A., Rotroff D.M., Freeman K., Clewell H.J., Dix D.J., Andersen M.E., Houck K.A. (2012). Integration of dosimetry, exposure, and high-throughput screening data in chemical toxicity assessment. Toxicol. Sci..

[33] Rajagopal R., Baltazar M.T., Carmichael P.L., Dent M.P., Head J., Li H., Muller I., Reynolds J., Sadh K., Simpson W. (2022). Beyond AOPs: A Mechanistic Evaluation of NAMs in DART Testing. Front. Toxicol..

[34] van der Burg B., Wedebye E.B., Dietrich D.R., Jaworska J., Mangelsdorf I., Paune E., Schwarz M., Piersma A.H., Kroese E.D. (2015). The ChemScreen project to design a pragmatic alternative approach to predict reproductive toxicity of chemicals. Reprod. Toxicol..

[35] Beilmann M., Boonen H., Czich A., Dear G., Hewitt P., Mow T., Newham P., Oinonen T., Pognan F., Roth A. (2019). Optimizing drug discovery by Investigative Toxicology: Current and future trends. ALTEX.

[36] Li J., Settivari R., LeBaron M.J., Marty M.S. (2019). An industry perspective: A streamlined screening strategy using alternative models for chemical assessment of developmental neurotoxicity. Neurotoxicology.

[37] Bal-Price A., Pistollato F., Sachana M., Bopp S.K., Munn S., Worth A. (2018). Strategies to improve the regulatory assessment of developmental neurotoxicity (DNT) using in vitro methods. Toxicol. Appl. Pharmacol..

[38] Leist M., Ghallab A., Graepel R., Marchan R., Hassan R., Bennekou S.H., Limonciel A., Vinken M., Schildknecht S., Waldmann T. (2017). Adverse outcome pathways: Opportunities, limitations and open questions. Arch. Toxicol..

[39] Mica Y., Lee G., Chambers S.M., Tomishima M.J., Studer L. (2013). Modeling neural crest induction, melanocyte specification, and disease-related pigmentation defects in hESCs and patient-specific iPSCs. Cell Rep..

[40] Zimmer B., Lee G., Balmer N.V., Meganathan K., Sachinidis A., Studer L., Leist M. (2012). Evaluation of developmental toxicants and signaling pathways in a functional test based on the migration of human neural crest cells. Environ. Health Perspect..

[41] Stiegler N.V., Krug A.K., Matt F., Leist M. (2011). Assessment of chemical-induced impairment of human neurite outgrowth by multiparametric live cell imaging in high-density cultures. Toxicol. Sci..

[42] Krebs A., Nyffeler J., Karreman C., Schmidt B.Z., Kappenberg F., Mellert J., Pallocca G., Pastor M., Rahnenführer J., Leist M. (2020). Determination of benchmark concentrations and their statistical uncertainty for cytotoxicity test data and functional in vitro assays. ALTEX.

[43] Delp J., Funke M., Rudolf F., Cediel A., Bennekou S.H., van der Stel W., Carta G., Jennings P., Toma C., Gardner I. (2019). Development of a neurotoxicity assay that is tuned to detect mitochondrial toxicants. Arch. Toxicol..

[44] Khalidi H., Onasanwo A., Islam B., Jo H., Fisher C., Aidley R., Gardner I., Bois F.Y. (2022). SimRFlow: An R-based workflow for automated high-throughput PBPK simulation with the Simcyp^®^ simulator. Front. Pharmacol..

[45] Rodgers T., Rowland M. (2006). Physiologically based pharmacokinetic modelling 2: Predicting the tissue distribution of acids, very weak bases, neutrals and zwitterions. J. Pharm. Sci..

[46] Kilford P.J., Gertz M., Houston J.B., Galetin A. (2008). Hepatocellular binding of drugs: Correction for unbound fraction in hepatocyte incubations using microsomal binding or drug lipophilicity data. Drug Metab. Dispos..

[47] Sugano K. (2009). Theoretical investigation of passive intestinal membrane permeability using Monte Carlo method to generate drug-like molecule population. Int. J. Pharm..

[48] Pade D., Jamei M., Rostami-Hodjegan A., Turner D.B. (2017). Application of the MechPeff model to predict passive effective intestinal permeability in the different regions of the rodent small intestine and colon. Biopharm. Drug Dispos..

[49] Sugano K. (2009). Computational oral absorption simulation for low-solubility compounds. Chem. Biodivers..

[50] Abduljalil K., Badhan R.K.S. (2020). Drug dosing during pregnancy-opportunities for physiologically based pharmacokinetic models. J. Pharmacokinet. Pharmacodyn..

[51] Abduljalil K., Furness P., Johnson T.N., Rostami-Hodjegan A., Soltani H. (2012). Anatomical, physiological and metabolic changes with gestational age during normal pregnancy: A database for parameters required in physiologically based pharmacokinetic modelling. Clin. Pharmacokinet..

[52] Ke A.B., Greupink R., Abduljalil K. (2018). Drug Dosing in Pregnant Women: Challenges and Opportunities in Using Physiologically Based Pharmacokinetic Modeling and Simulations. CPT Pharmacometrics. Syst. Pharmacol..

[53] Lu G., Abduljalil K., Jamei M., Johnson T.N., Soltani H., Rostami-Hodjegan A. (2012). Physiologically-based pharmacokinetic (PBPK) models for assessing the kinetics of xenobiotics during pregnancy: Achievements and shortcomings. Curr. Drug. Metab..

[54] Abduljalil K., Jamei M., Johnson T.N. (2019). Fetal Physiologically Based Pharmacokinetic Models: Systems Information on the Growth and Composition of Fetal Organs. Clin. Pharmacokinet..

[55] Abduljalil K., Jamei M., Johnson T.N. (2020). Fetal Physiologically Based Pharmacokinetic Models: Systems Information on Fetal Blood Components and Binding Proteins. Clin. Pharmacokinet..

[56] Abduljalil K., Johnson T.N., Rostami-Hodjegan A. (2018). Fetal Physiologically-Based Pharmacokinetic Models: Systems Information on Fetal Biometry and Gross Composition. Clin. Pharmacokinet..

[57] Abduljalil K., Pan X., Clayton R., Johnson T.N., Jamei M. (2021). Fetal Physiologically Based Pharmacokinetic Models: Systems Information on Fetal Cardiac Output and Its Distribution to Different Organs during Development. Clin. Pharmacokinet..

[58] Abduljalil K., Ning J., Pansari A., Pan X., Jamei M. (2022). Prediction of Maternal and Fetoplacental Concentrations of Cefazolin, Cefuroxime, and Amoxicillin during Pregnancy Using Bottom-Up Physiologically Based Pharmacokinetic Models. Drug Metab. Dispos..

[59] Fisher C., Siméon S., Jamei M., Gardner I., Bois Y.F. (2019). VIVD: Virtual in vitro distribution model for the mechanistic prediction of intracellular concentrations of chemicals in in vitro toxicity assays. Toxicol. In Vitro.

[60] Delp J., Cediel-Ulloa A., Suciu I., Kranaster P., van Vugt-Lussenburg B.M., Munic Kos V., van der Stel W., Carta G., Bennekou S.H., Jennings P. (2021). Neurotoxicity and underlying cellular changes of 21 mitochondrial respiratory chain inhibitors. Arch. Toxicol..

[61] van der Stel W., Carta G., Eakins J., Darici S., Delp J., Forsby A., Bennekou S.H., Gardner I., Leist M., Danen E.H.J. (2020). Multiparametric assessment of mitochondrial respiratory inhibition in HepG2 and RPTEC/TERT1 cells using a panel of mitochondrial targeting agrochemicals. Arch. Toxicol..

[62] Hallinger D.R., Lindsay H.B., Friedman K.P., Suarez D.A., Simmons S.O. (2020). Respirometric Screening and Characterization of Mitochondrial Toxicants Within the ToxCast Phase I and II Chemical Libraries. Toxicol. Sci..

[63] Leist M., Single B., Castoldi A.F., Kühnle S., Nicotera P. (1997). Intracellular adenosine triphosphate (ATP) concentration: A switch in the decision between apoptosis and necrosis. J. Exp. Med..

[64] Volbracht C., Leist M., Nicotera P. (1999). ATP controls neuronal apoptosis triggered by microtubule breakdown or potassium deprivation. Mol. Med..

[65] Pöltl D., Schildknecht S., Karreman C., Leist M. (2012). Uncoupling of ATP-depletion and cell death in human dopaminergic neurons. Neurotoxicology.

[66] Mookerjee S.A., Gerencser A.A., Nicholls D.G., Brand M.D. (2017). Quantifying intracellular rates of glycolytic and oxidative ATP production and consumption using extracellular flux measurements. J. Biol. Chem..

[67] Mookerjee S.A., Goncalves R.L.S., Gerencser A.A., Nicholls D.G., Brand M.D. (2015). The contributions of respiration and glycolysis to extracellular acid production. Biochim. Biophys. Acta.

[68] Desousa B.R., Kim K.K., Jones A.E., Ball A.B., Hsieh W.Y., Swain P., Morrow D.H., Brownstein A.J., Ferrick D.A., Shirihai O.S. (2023). Calculation of ATP production rates using the Seahorse XF Analyzer. EMBO Rep..

[69] Terron A., Bal-Price A., Paini A., Monnet-Tschudi F., Bennekou S.H., Members E.W.E., Leist M., Schildknecht S. (2018). An adverse outcome pathway for parkinsonian motor deficits associated with mitochondrial complex I inhibition. Arch. Toxicol..

[70] Shi N.-N., Lian J.-P., Qiu D.-Z., Chen F.-R., Du Y.-X. (2023). Resistance risk and molecular mechanism associated with resistance to picoxystrobin in *Colletotrichum truncatum* and *Colletotrichum gloeosporioides*. J. Integr. Agric..

[71] (2016). European Food Safety Authority. A Peer review of the pesticide risk assessment of the active substance picoxystrobin. EFSA J..

[72] OECD (2007). Test No. 426: Developmental Neurotoxicity Study. OECD Guidelines for the Testing of Chemicals, Section 4.

[73] OECD (2023). Initial Recommendations on Evaluation of Data from the Developmental Neurotoxicity (DNT) In-Vitro Testing Battery.

[74] Vincent F., Loria P.M., Weston A.D., Steppan C.M., Doyonnas R., Wang Y.M., Rockwell K.L., Peakman M.C. (2020). Hit Triage and Validation in Phenotypic Screening: Considerations and Strategies. Cell Chem. Biol..

[75] Vincent F., Nueda A., Lee J., Schenone M., Prunotto M., Mercola M. (2022). Phenotypic drug discovery: Recent successes, lessons learned and new directions. Nat. Rev. Drug. Discov..

[76] Chaput L., Guillaume V., Singh N., Deprez B., Villoutreix B.O. (2020). FastTargetPred: A program enabling the fast prediction of putative protein targets for input chemical databases. Bioinformatics.

[77] Suciu I., Delp J., Gutbier S., Suess J., Henschke L., Celardo I., Mayer T.U., Amelio I., Leist M. (2023). Definition of the Neurotoxicity-Associated Metabolic Signature Triggered by Berberine and Other Respiratory Chain Inhibitors. Antioxidants.

[78] Meier M.J., Harrill J., Johnson K., Thomas R.S., Tong W., Rager J.E., Yauk C.L. (2024). Progress in toxicogenomics to protect human health. Nat. Rev. Genet..

[79] Smirnova L., Hogberg H.T., Leist M., Hartung T. (2024). Revolutionizing developmental neurotoxicity testing—A journey from animal models to advanced in vitro systems. ALTEX.

[80] Thomas R.S., Bahadori T., Buckley T.J., Cowden J., Deisenroth C., Dionisio K.L., Frithsen J.B., Grulke C.M., Gwinn M.R., Harrill J.A. (2019). The Next Generation Blueprint of Computational Toxicology at the U.S. Environ. Prot. Agency. Toxicol. Sci..

[81] Blaauboer B.J., Boobis A.R., Bradford B., Cockburn A., Constable A., Daneshian M., Edwards G., Garthoff J.A., Jeffery B., Krul C. (2016). Considering new methodologies in strategies for safety assessment of foods and food ingredients. Food Chem. Toxicol..

[82] Pallocca G., Moné M.J., Kamp H., Luijten M., van de Water B., Leist M. (2022). Next-generation risk assessment of chemicals—Rolling out a human-centric testing strategy to drive 3R implementation: The RISK-HUNT3R project perspective. ALTEX.

[83] Berggren E., White A., Ouedraogo G., Paini A., Richarz A.N., Bois F.Y., Exner T., Leite S., Grunsven L.A.V., Worth A. (2017). Ab initio chemical safety assessment: A workflow based on exposure considerations and non-animal methods. Comput. Toxicol..

[84] Dent M.P., Vaillancourt E., Thomas R.S., Carmichael P.L., Ouedraogo G., Kojima H., Barroso J., Ansell J., Barton-Maclaren T.S., Bennekou S.H. (2021). Paving the way for application of next generation risk assessment to safety decision-making for cosmetic ingredients. Regul. Toxicol. Pharmacol..

[85] Bell S.M., Chang X., Wambaugh J.F., Allen D.G., Bartels M., Brouwer K.L.R., Casey W.M., Choksi N., Ferguson S.S., Fraczkiewicz G. (2018). In vitro to in vivo extrapolation for high throughput prioritization and decision making. Toxicol. In Vitro.

[86] Chang X., Tan Y.M., Allen D.G., Bell S., Brown P.C., Browning L., Ceger P., Gearhart J., Hakkinen P.J., Kabadi S.V. (2022). IVIVE: Facilitating the Use of In Vitro Toxicity Data in Risk Assessment and Decision Making. Toxics.

