# Supplementary material for: Inhibition of Neural Crest Cell Migration by Strobilurin Fungicides and Other Mitochondrial Toxicants
Source: Cells. 2024 Dec 12;13(24):2057. doi: 10.3390/cells13242057 (PMC11674305; doi:10.3390/cells13242057)
Supplement: Supplementary file 1 [file cells-13-02057-s001.zip › cells-3327189-supplementary.pdf]

Supplementary information for

Inhibition of neural crest cell migration by strobilurin fungicides and other mitochondrial toxicants

Viktoria Magel[1, †], Jonathan Blum[1, †], Xenia Dolde[1], Heidrun Leisner[1], Karin Grillberger[2], Hiba Khalidi[3], Iain Gardner[3], Gerhard F. Ecker[2], Giorgia Pallocca[1,4], Nadine Dreser[1,4] & Marcel Leist[1,4,*]

[1] In Vitro Toxicology and Biomedicine, Dept Inaugurated by the Doerenkamp-Zbinden Foundation, University of Konstanz, 78464 Konstanz, Germany
[2] Department of Pharmaceutical Chemistry, University of Vienna, 1090 Vienna, Austria
[3] Certara Predictive Technologies, Level 2-Acero, 1 Concourse Way, Sheffield, S1 2BJ, UK
[4] CAAT-Europe, University of Konstanz, 78464 Konstanz, Germany
*Correspondence: marcel.leist@uni-konstanz.de; Tel.: +49-(0)-7531-88-5037
[†] These authors contributed equally to this work.

Table of Contents

**Figure S1**	cMINC (UKN2) exposure scheme and imaging exemplification	page 2
**Figure S2**	Results of Pre-Screen 1	page 3
**Figure S3**	Pre-Screen 1 synopsis of results	page 4
**Figure S4**	Overview of results from Pre-Screen 2	page 5
**Figure S5**	Exemplary primary screen hits	page 6
**Figure S6**	ATP level after exposure to some mitochondrial toxicants	page 7
**Figure S7**	Confirmation testing of picoxystrobin using a new stock	page 8
**Figure S8**	Docking of picoxystrobin at complex III	page 9
**Figure S9**	Inhibition of electron transport chain complexes by picoxystrobin in neural crest cells	page 10
**Figure S10**	Modelling of maternal brain concentrations of picoxystrobin	page 11
**Figure S11**	Prolonged exposure of NCC in a modified transwell assay setup	page 12

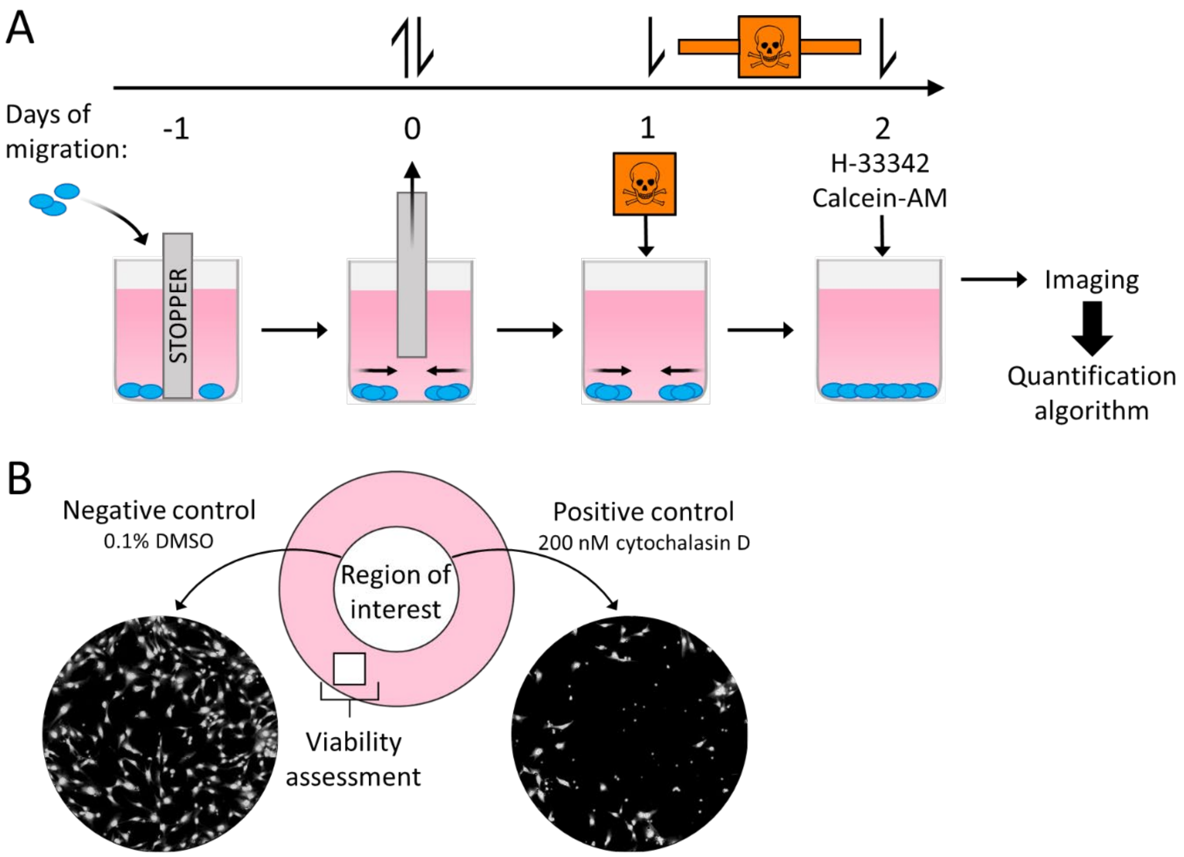

Figure S1. cMINC (UKN2) exposure scheme and imaging exemplification.

(A) To prepare for the assay, neural crest cells were seeded around a stopper on day of migration (DoM) -1. Stoppers were removed after 24 h on DoM 0 to create a cell-free area. The removal of the stoppers initiates migration into the cell-free area. On DoM 1, cells were exposed for 24 h to the tested compounds. At 24 h later (DoM 2), cells were stained with calcein-AM and H-33342 to perform high-content imaging. **(B)** The region of interest (previously area of the stopper) was imaged to assess migration. Exemplary pictures of the negative control (0.1% DMSO) on the left and the positive control (200 nM cytochalasin D (CytoD)) on the right side are shown. To assess the viability, four images were taken outside the migration area, and calcein H-33342 double-positive cell numbers and fractions were determined by an automated algorithm.

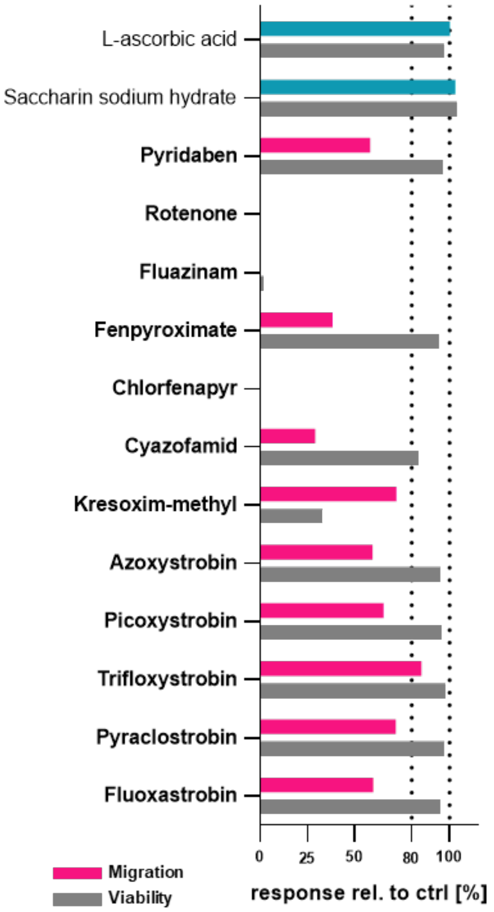

Figure S2. Results of Pre-Screen 1.

All library compounds were tested (four technical replicates) at a concentration 1:1000 of the stock. The final test concentration corresponded in most cases to 100 µM. The mitochondrial inhibitors, which showed an effect in the pre-screen 1, and two negative controls are displayed. The dotted line at 100% indicates the "no effect level". The dotted line at 80% indicates the 20% benchmark response (BMR20) which was the threshold defined for reduced viability and migration. Compounds were selected for pre-screen-2, if migration or viability were below 80%. Compounds close to the 80% limit were also included to avoid false negatives. Moreover, some randomly picked negatives were included to test specificity of pre-screen 2. All values are normalized to the solvent control (0.1% DMSO). 1N (each 4n).

Compound	Migration	Viability
L-ascorbic acid	100	97
Saccharin sodium hydrate	103	104
Pyridaben	58	96
Rotenone	0	0
Fluazinam	0	2
Fenpyroximate	38	95
Chlorfenapyr	0	0
Cyazofamid	29	84
Kresoxim-methyl	72	33
Azoxystrobin	59	95
Picoxystrobin	65	96
Trifloxystrobin	85*	98
Pyraclostrobin	72	97
Fluoxastrobin	60	95

Figure S3. Pre-Screen 1 synopsis of results.

Overview of Pre-Screen 1 results. Data are given in % of control. Migration of compounds highlighted in pink was below the threshold of 80%. They were followed up in Pre-Screen 2. Trifloxystrobin was also included, as migration was close to the threshold (with unimpaired viability).

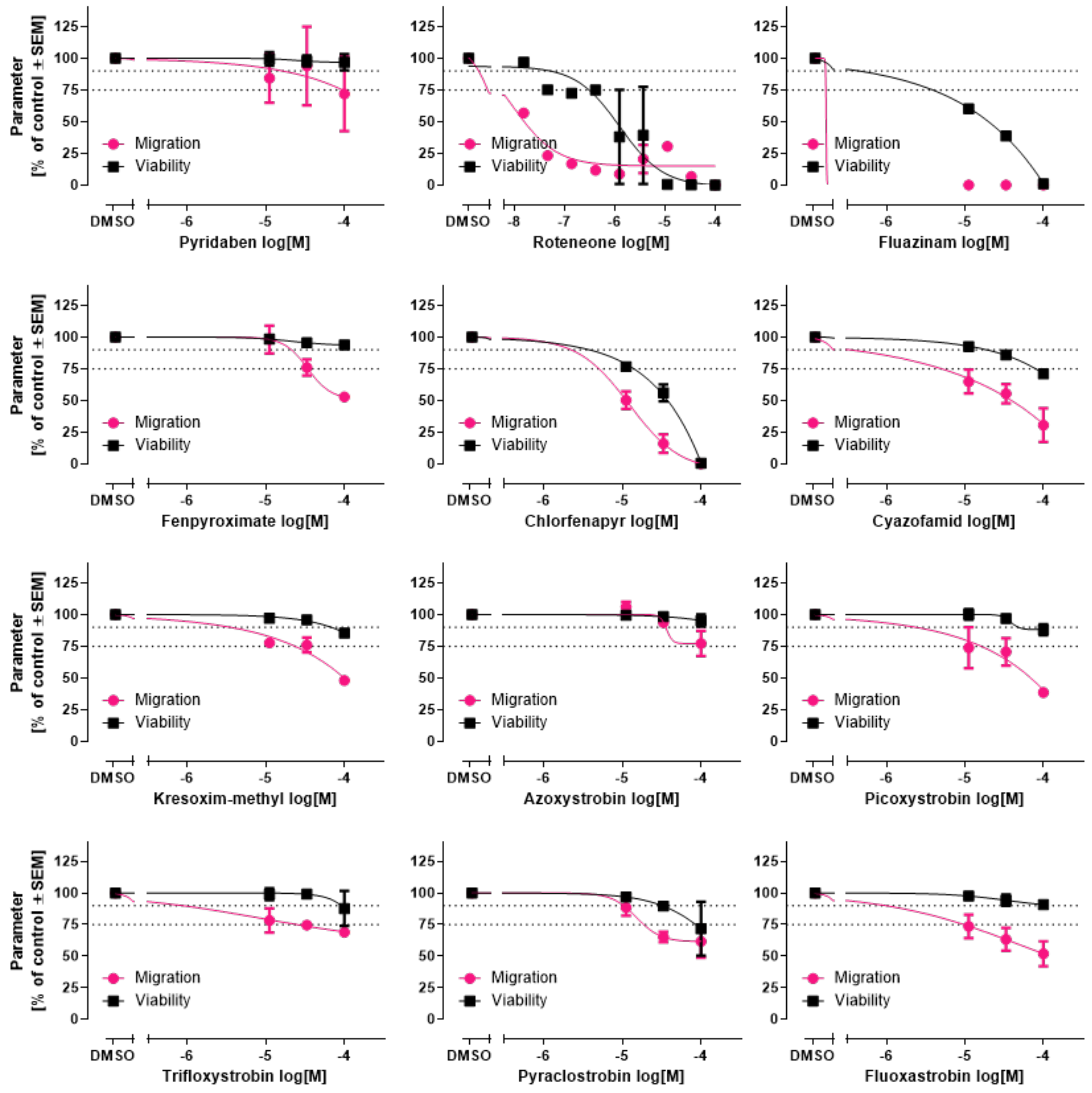

Figure S4. Overview of results from Pre-Screen 2.

Mitochondrial inhibitors identified in Pre-Screen 1 were followed up in Pre-Screen 2 with at least 3 test concentrations. The compounds displayed in the figure had a reduced migration by ≥20%. They also affected migration more potently than viability. They were defined as "preliminary hit pool" and they were subsequently followed up in the primary screen. At least 2N (each 3n).

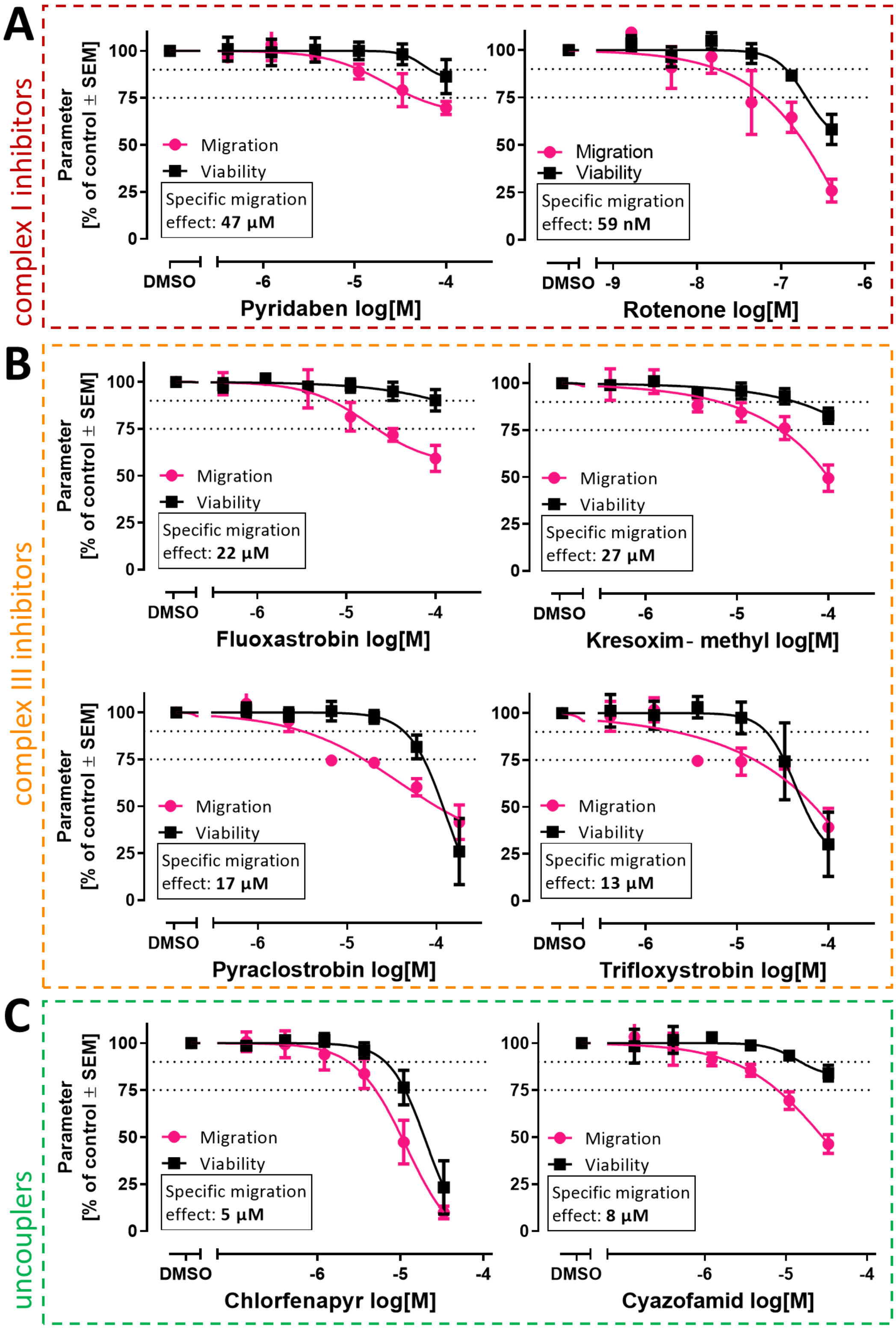

Figure S5. Exemplary primary screen hits.

The graphs show the results of the primary screen of some mitochondrial toxicants. They are grouped as **(A)** complex I-inhibitors (red); **(B)** the strobilurins group, complex III-inhibitors (orange); **(C)** uncouplers (green). This supplementary figure contains data on compounds not displayed in main Fig. 3. Summary data are based on ≥ 3N (each 3n).

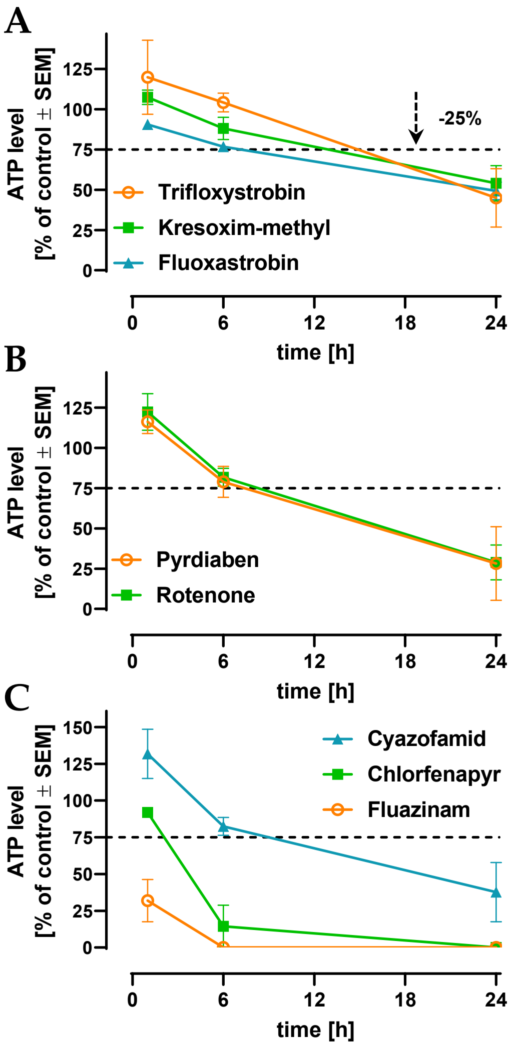

Figure S6. ATP levels after exposure to some mitochondrial toxicants.

NCC were exposed with the BMC10(V) concentration of the cMINC screen. The graphs show ATP levels after treatment of NCC with mitochondrial toxicants for 1 h, 6 h and 24 h relative to the solvent control. **(A)** Complex III – inhibitors; **(B)** Complex I – inhibitors; **(C)** Uncouplers. This supplementary figure contains data on compounds not shown in main Figure 3. Summary data are based on 3N (each 3n).

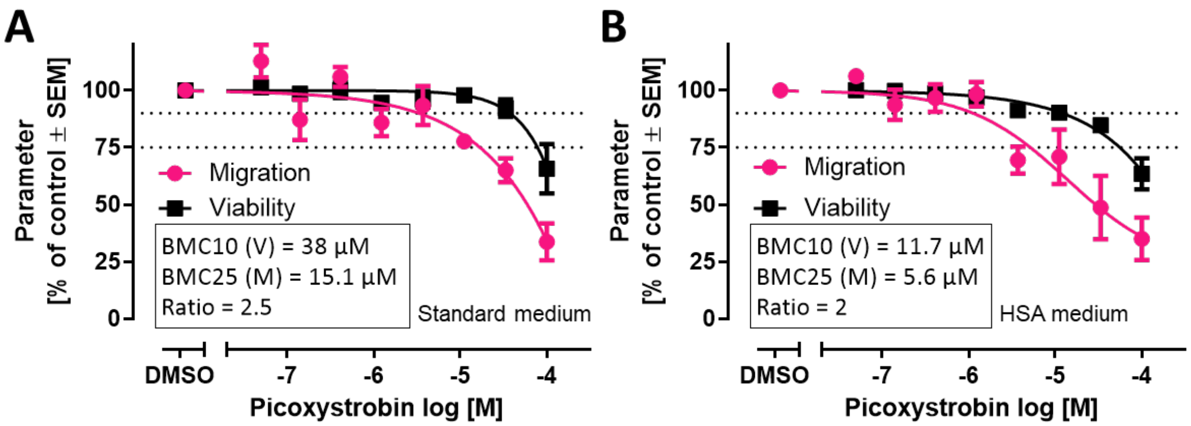

Figure S7. Confirmation testing of picoxystrobin using a new stock.

The graphs show the results of the cMINC assay for which a new stock of picoxystrobin was tested to reconfirm the screen results. **(A)** NCC migration and viability were re-assessed by a different operator. **(B)** To verify that picoxystrobin affects NCC migration also in the presence of Human Serum Albumin (HSA), the medium was supplemented with 1% HSA.

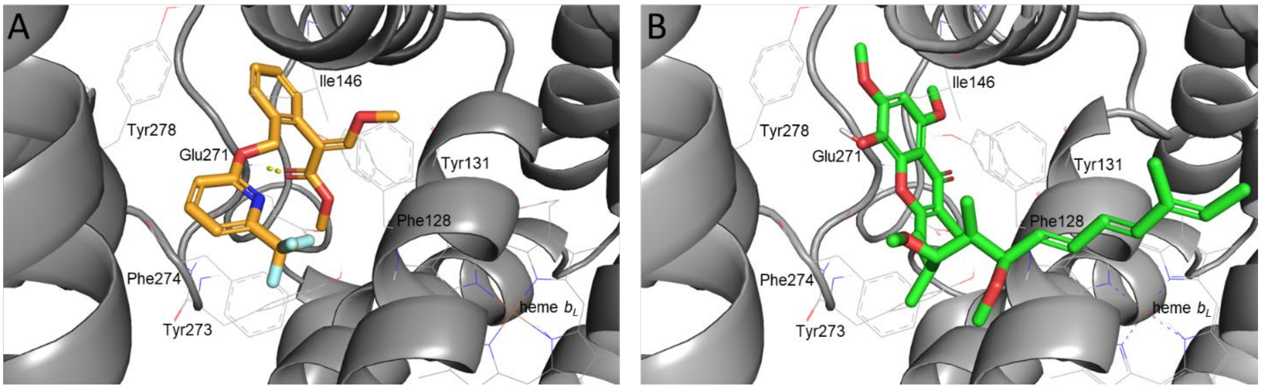

Figure S8: Docking of picoxystrobin at complex III.

The human apo-protein structure of mitochondrial complex III (cytochrome bc1 complex, PDB-ID: 5xte) was extracted from the protein data bank (Berman *et al.*, 2000; Guo *et al.*, 2017), and chain V was used for the docking study. Both, protein and ligands were modeled at pH 7.4 ± 0.5 using "Protein preparation Wizard" and "Ligprep" provided by Schrödinger, LLC (Schrödinger Release 2021-1: Protein Preparation Wizard; Epik, Schrödinger, LLC, New York, NY, 2021), following the methodologies described by Sastry and colleagues (Sastry *et al.*, 2013). The docking software GOLD (Jones *et al.*, 1997) was used for a flexible docking protocol. The grid center was defined by the coordinates X:226.08; Y:319.31; Z: 232.34. Then, 100 genetic algorithm (GA) runs were performed per ligand (i.e. 100 docking poses were generated). The following residues were set as flexible sidechains: Phe128, Tyr131, Met138, Ile146, Glu271, Tyr273, Phe274, Tyr278, and Leu281. The top two docking poses were selected based on their piecewise linear potential (PLP) fitness scores and visual inspection. The PLP fitness score evaluates the quality of generated poses by assessing the attractive and repulsive forces within the protein-ligand complex. Visualization was performed in Pymol 2.5 (Schrödinger, LLC, 2021). **(A)** The docking pose of picoxystrobin (orange carbon atoms) is shown. Picoxystrobin forms a hydrogen bond to Glu271, which is also reported from the literature (Esser *et al.*, 2004). **(B)** For comparison, the docking pose of stigmatellin (green carbon atoms) is shown, which is also a specific binder at this site (Huang *et al.*, 2005). Both compounds bind to the Q_o binding site, in vicinity of the low-potential heme (heme b_L) of the human structure of mitochondrial complex III (cytochrome bc_1 complex).

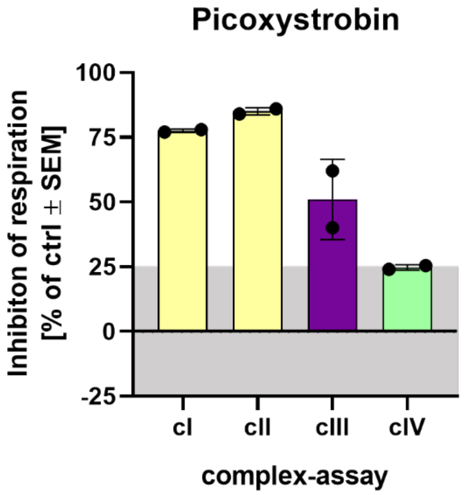

Figure S9. Inhibition of electron transport chain complexes by picoxystrobin in neural crest cells.

NCC were exposed to the BMC10(V) of the cMINC assay (60 µM). The grey area indicates the non-significant range of the assay (≤ 25% change). The effect on complex IV (cIV) was non-significant (green bar). The purple bar indicates complex III (cIII) inhibition. Yellow bars indicate an "apparent inhibition" of complex I (cI) and complex II (cII). This is due to the fact that for these assays, the electrons have to pass cIII to reach cIV (oxygen consumption), i.e. electrons from cI (or cII) cannot be used for the reduction of oxygen, as they need to flow through cIII (which is blocked). The pattern of inhibition is indicative of cIII as illustrated earlier (Delp *et al.*, 2019; van der Stel *et al.*, 2020).

To investigate the inhibition pattern of picoxystrobin, NCC were permeabilized and complex-specific substrates and inhibitors were sequentially injected as described earlier (Delp *et al.*, 2019). In detail, cells were permeabilized with MAS buffer (220 mM mannitol, 1 mM ADP, 70 mM sucrose, 10 mM KH2PO4, 5 mM MgCl2, 2 mM HEPES, 1 mM EGTA, 4 mg/ml fatty acid-free BSA, pH = 7.2) supplemented with 25 µg/mL digitonin. Seahorse measurements were started directly afterwards, and basal OCRs were assessed for internal normalization. Then picoxystrobin was injected and cI activity was assessed. For this, cII was inhibited (5 mM malonat) and substrates for cI (2 mM L-Glutamine, 2.5 mM malic acid, 5 mM pyruvic acid) were added. Apparent inhibition of picoxystrobin was observed in this step. Next, cI was inhibited (0.5 µM rotenone) and substrates of cIII (250 µM duroquinol) were added. At this step, cIII inhibition data for cIII were obtained. In the case of a cI inhibition (e.g. by rotenone), oxygen consumption rate would have increased after addition of duroquinol (but it did not). To assess cII activity, cI was inhibited and the substrate of cII (10 mM succinic acid) was added. Lastly, to assess cIV activity, cII was inhibited and the substrates of cIV (125 µM TMPD, 2 mM ascorbic acid) were added. Data were compared to the solvent control. Details and assay validation have been provided earlier (Delp *et al.*, 2019; van der Stel *et al.*, 2020). Data are from 2N.

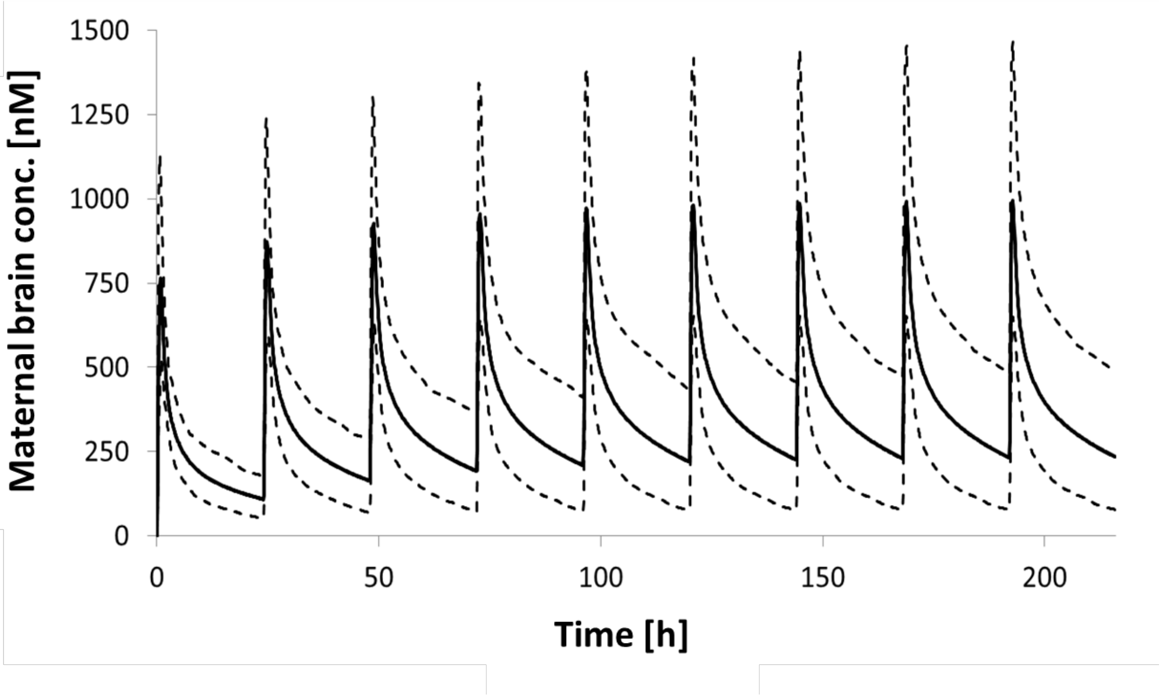

Figure S10. Modelling of maternal brain concentrations of picoxystrobin.

A physiologically-based kinetics (PBK) model was established for picoxystrobin. The model was parametrized to reflect human subjects after a daily oral dose of 0.09 mg/kg. The predicted concentrations of picoxystrobin are shown. Data (solid line) are population averages of subjects (n = 100), aged between 20-50. The dashed lines indicate the 95 % confidence intervals.

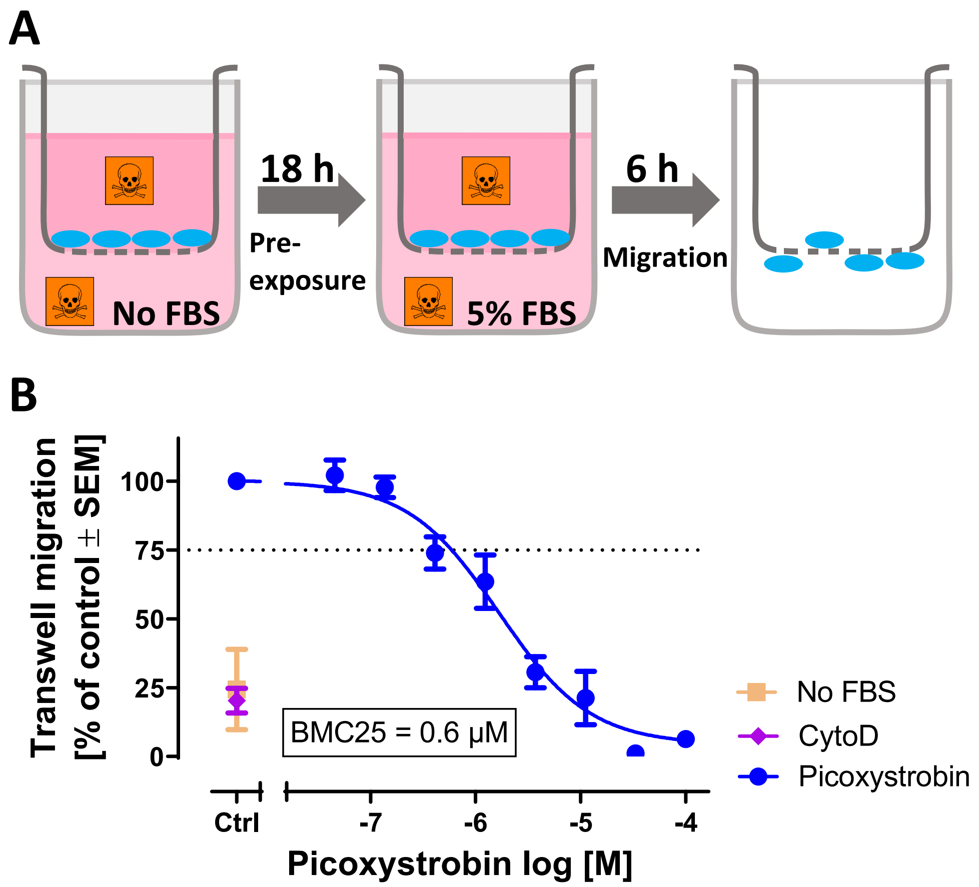

Figure S11. Prolonged exposure of NCC in a modified transwell assay setup.

(A) Schematic illustration of the "long-term" transwell migration assay. In the beginning, NCC were plated into the insert of the transwell and treated with potential toxicants. In comparison to the standard transwell set-up, the lower medium compartment was initially not supplemented with FBS. In order to ensure a uniform concentration, picoxystrobin was added to both compartments. Cells were then incubated for 18 h (in the absence of a migration stimulus). Afterwards, 5% FBS was added to the lower compartment, to initiate NCC migration through the membrane pores. After 6 h, the number of cells that reached the downward surface of the membrane was quantified. **(B)** Result of concentration-dependent picoxystrobin testing in galactose medium in the long-term transwell assay. Cytochalasin D (200 nM) was used as positive control for "inhibited" migration (CytoD). "Unstimulated" migration (no FBS) is also shown. The dotted line at 75% indicates the threshold for inhibited migration. Data are expressed as means ± SEM from 3 independent biological experiments.

Supplementary References

Berman, H. M., Westbrook, J., Feng, Z., Gilliland, G., Bhat, T. N., Weissig, H., Shindyalov, I. N. and Bourne, P. E. (2000) 'The Protein Data Bank', *Nucleic Acids Research,* 28(1), pp. 235-242.

Delp, J., Funke, M., Rudolf, F., Cediel, A., Bennekou, S. H., van der Stel, W., Carta, G., Jennings, P., Toma, C., Gardner, I., van de Water, B., Forsby, A. and Leist, M. (2019) 'Development of a neurotoxicity assay that is tuned to detect mitochondrial toxicants', *Arch Toxicol,* 93(6), pp. 1585-1608.

Esser, L., Quinn, B., Li, Y.-F., Zhang, M., Elberry, M., Yu, L., Yu, C.-A. and Xia, D. (2004) 'Crystallographic Studies of Quinol Oxidation Site Inhibitors: A Modified Classification of Inhibitors for the Cytochrome bc1 Complex', *Journal of Molecular Biology,* 341(1), pp. 281-302.

Guo, R., Zong, S., Wu, M., Gu, J. and Yang, M. (2017) 'Architecture of Human Mitochondrial Respiratory Megacomplex I2III2IV2', *Cell,* 170(6), pp. 1247-1257.e12.

Huang, L.-s., Cobessi, D., Tung, E. Y. and Berry, E. A. (2005) 'Binding of the Respiratory Chain Inhibitor Antimycin to the Mitochondrial bc1 Complex: A New Crystal Structure Reveals an Altered Intramolecular Hydrogen-bonding Pattern', *Journal of Molecular Biology,* 351(3), pp. 573-597.

Jones, G., Willett, P., Glen, R. C., Leach, A. R. and Taylor, R. (1997) 'Development and validation of a genetic algorithm for flexible docking11Edited by F. E. Cohen', *Journal of Molecular Biology,* 267(3), pp. 727-748.

Sastry, G. M., Adzhigirey, M., Day, T., Annabhimoju, R. and Sherman, W. (2013) 'Protein and ligand preparation: parameters, protocols, and influence on virtual screening enrichments', *J Comput Aided Mol Des,* 27(3), pp. 221-34.

van der Stel, W., Carta, G., Eakins, J., Darici, S., Delp, J., Forsby, A., Bennekou, S. H., Gardner, I., Leist, M., Danen, E. H. J., Walker, P., van de Water, B. and Jennings, P. (2020) 'Multiparametric assessment of mitochondrial respiratory inhibition in HepG2 and RPTEC/TERT1 cells using a panel of mitochondrial targeting agrochemicals', *Arch Toxicol,* 94(8), pp. 2707-2729.

Schrödinger Release 2021-1: LigPrep, 2021.

Schrödinger Release 2021-1: Protein Preparation Wizard; Epik, 2021.
